# The role of auxin transport through plasmodesmata in leaf vein canalization and patterning

**DOI:** 10.3389/fpls.2025.1621815

**Published:** 2025-10-01

**Authors:** David M. Holloway, Trausti K. Eiriksson, Carol L. Wenzel

**Affiliations:** ^1^ Mathematics Department, British Columbia Institute of Technology, Burnaby, BC, Canada; ^2^ Chemistry Department, University of Toronto, Toronto, ON, Canada; ^3^ Biotechnology Department, British Columbia Institute of Technology, Burnaby, BC, Canada

**Keywords:** auxin, canalization, leaf venation, mathematical modelling, PIN, plasmodesmata, polar auxin transport, vascular

## Abstract

Vein patterns in plant leaves are preceded by high concentration localized tracks of the phytohormone auxin. Auxin regulates downstream genes involved in vascular differentiation. Proposals for the mechanisms by which auxin canalizes from broad early distributions to later narrow provascular tracks have been made for many decades and tested in mathematical models. These have focused on PIN1, a membrane-bound protein involved in exporting auxin from cells. *PIN* mutations and interference with polar auxin transport (PAT) through PIN have strong effects on vein patterns. However, recent experiments show that even with PIN-dependent PAT presumably shut off, veins form and extend, albeit with altered patterning. This residual canalization and vein patterning has a dependence on flow through plasmodesmata (PD) intercellular channels. We developed a new mathematical framework for the regulation of auxin flow through both PIN and PD. This produces better fits to data than prior PIN-only models, especially with respect to vein number, directionality and extension in reduced PIN transport conditions. Varying PD area recapitulates known experimental results with PD mutants, in particular the loss of canalization at high PD permeability. Model parameters are consistent with measured permeabilities and predict effects for future experiments. This work updates the canalization hypothesis for auxin provascular strand formation in early leaf development in terms of the contributions from both PIN and PD.

## 1 Introduction

**Figure 1:**
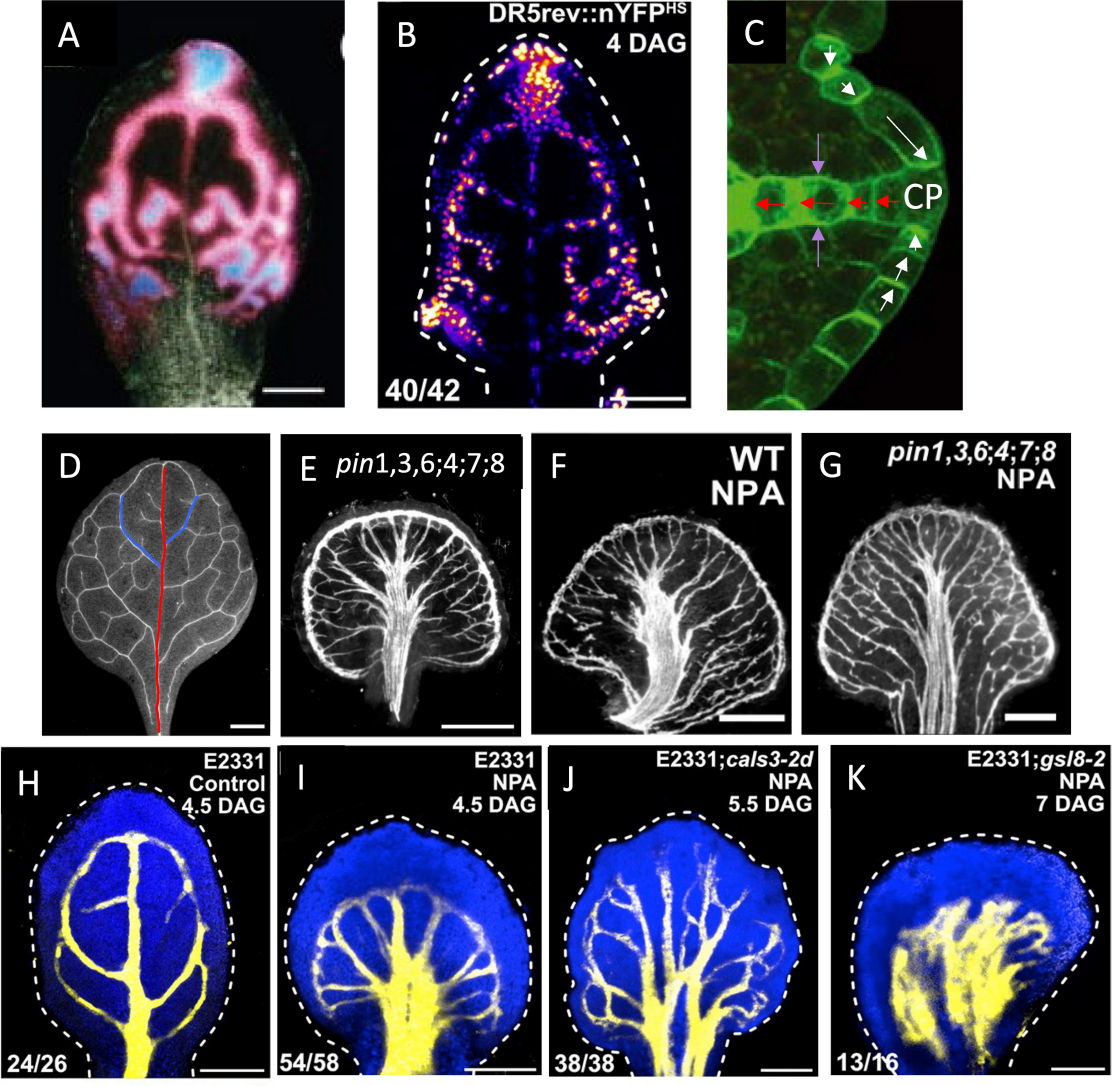
Auxin transport in vein patterning. **(A, B)** Auxin forms provascular tracks in the locations of the future veins, flowing from sources at the margin towards the leaf centre and base. **(A)** DR5::GUS auxin response marker, indicated by pink-low and blue-high levels (from Mattsson et al., 2003, with permission); **(B)** DR5rev::nYFP^ES^, yellow-high and blue-low levels (from Linh and Scarpella, 2022, with permission). **(C)** Polar auxin transport (PAT) through PIN1 (green) membrane-bound auxin exporters facilitates flow both away from (red arrows) and towards (white arrows) high auxin sources (CP, convergence points; from Wenzel et al., 2007, with permission); purple arrows indicate toward-vein alignment of PIN1 along the lateral walls. Interference with auxin transport through PIN, either through multiple *PIN* mutations, such as *pin*1,3,6;4;7;8 **(E)**, or by treatment with the drug NPA **(F)** results in more secondary-like veins extending in from the margin, and alters the joining of veins, resulting in bundling in the centre, compared to WT (D, red line – primary vein; blue lines – earliest-formed secondary veins). Combined mutation and NPA treatment do not produce additional severity of the phenotype **(G)**, suggesting an underlying non-PIN vein patterning mechanism. **(D–G)** from Verna et al. (2019) with permission. **(H)** veins (yellow) visualized by YFP activation in a control leaf of enhancer trap line E2331. **(I–K)** Vein formation in E2331 under NPA treatment (reduced PIN transport): canalized veins form in E2331;WT **(I)** and with E2331;*cals3-2d* narrow-aperture PD **(J)**, but veins do not canalize with E2331;*gsl8–2* wide-aperture PD **(K)**. **(H–K)** from Linh and Scarpella (2022) with permission.

Spatial patterning of the vascular network during leaf development has fascinated biologists for decades. While a functional network of veins is critical to water and solute transport throughout the leaf, the spatial patterning of leaf veins varies widely and can correlate in species-specific ways with leaf morphology. The future location of leaf veins can first be seen in narrow high concentration tracks of the hormone auxin (**Figures 1A, B**). Auxin regulates genes involved in the subsequent differentiation into vascular tissue, for example in the regulation of *MP* (Przemeck et al., 1996; Hardtke and Berleth, 1998; Wenzel et al., 2007), which activates *ATHB8*, one of the earliest markers of preprocambial vascular fate (Donner et al., 2009; Marcos and Berleth, 2014; Scarpella, 2024). (See **Table 1** for a list of abbreviations.) A major focus for leaf vein patterning, therefore, has been on the mechanisms that determine the spatial localization of these provascular auxin tracks.

**Table 1:** List of abbreviations.

Abbreviation	Definition
ABCB	ATP-BINDING CASSETTE subtype B, auxin efflux transporters
ARF	AUXIN RESPONSE FACTOR
ATHB	ARABIDOPSIS THALIANA HOMEOBOX, early vascular marker
AUX1/LAX	AUXIN1/Like AUX1, auxin influx transporters
CALS	CALLOSE SYNTHASE, narrows PD
CP	convergence point
GSL	GLUCAN SYNTHASE-LIKE, widens PD
*MP*	*MONOPTEROS*, auxin target
NPA	naphthylphthalamic acid, inhibitor of auxin transport through PIN
PAT	polar auxin transport
PD	plasmodesmata
PDLP	PLASMODESMATA LOCATED PROTEIN
PIN	PIN-FORMED, auxin efflux transporters
PIN-PAT-i	inhibited flow of auxin through PIN
SHI/STY	SHORT INTERNODE/STYLISH, auxin synthesis
WT	wild-type
WTF	with-the-flux PIN allocation to the membrane
UTG	up-the-gradient PIN allocation to the membrane

Early experiments with wound recovery characterized the polarized nature of auxin flow, from sources to sinks (e.g. shoots to roots; Sachs, 1969). In leaves, auxin flow is generally from the leaf margin (corresponding to expression domains of auxin-synthesis regulators such as YUCCA and SHI/STY — Cheng et al., 2006, Cheng et al., 2007; Wang et al., 2011; Baylis et al., 2013; Zhang et al., 2020) towards the leaf base where the leaf vasculature connects to the vasculature of the rest of the plant. In *Arabidopsis*, the polarity of later-formed tertiary veins and above indicates a role for auxin synthesis throughout the leaf (Scarpella et al., 2006; Marcos and Berleth, 2014). However, the earlier-formed primary (mid-vein; **Figure 1D**, red) and secondary (**Figure 1D**, blue) veins appear more robust to mutations in auxin synthesis genes (Kneuper et al., 2021) or interference with auxin signaling (Linh and Scarpella, 2022), and early disruption of auxin flow increases levels of auxin and venation in the leaf margins (e.g., **Figure 1E**; Mattsson et al., 1999, Mattsson et al., 2003; Verna et al., 2019), indicating a marginal source for these veins.

Auxin flow has a positive feedback with tissue polarity (e.g. auxin velocities increase with the exogenous application of auxin; Sachs, 1975) implying that as auxin patterns develop from initially non-polarized tissues (e.g. the initial flow from a source), the auxin distribution will become increasingly canalized into narrow strands (Sachs, 1978; discussed further in Sachs, 1981, Sachs, 1991). Sachs (1968) noted that transport without such feedback, for example by simple diffusion, would result in a hemispherical spreading of signal from a source, rather than the observed provascular strands.

The dynamic and adaptable nature of auxin patterning and canalization indicates that these processes are self-organizing, rather than being dictated by an upstream prepattern (e.g. see Sachs, 1991; Mazur et al., 2020). Increasing data on the molecular components involved and mathematical modelling of the dynamics of auxin flow and regulation both contribute to understanding how molecular and cellular scale processes distribute molecules into vein network patterns at the orders-of-magnitude larger scale of tissues.

Sachs’ original intuitive ‘canalization hypothesis’ was formalized into a mathematical model by Mitchison (1980, 1981), which demonstrated that a self-enhancing flow could indeed produce the canalizing tracks characteristic of provascular auxin patterns in leaves. See also Lavania et al. (2021) and Scarpella (2023, 2024) for recent reviews on leaf vein canalization.

Mitchison made two proposals for the canalization mechanism, both dependent on the intercellular auxin flux *ϕ*: Type 1 (Mitchison, 1980) was based on intercellular diffusion, with flux *ϕ* feeding back on and enhancing the auxin diffusivity between cells (also referred to as facilitated diffusion); Type 2 (Mitchison, 1981) was based on flow through transmembrane transporters, with the permeability through the transporters dependent on flux *ϕ*. He showed that for auxin concentration in a strand to drop with increasing flow (i.e. as the strand becomes more conductive) and for canalization into tracks to occur, the diffusivity or permeability needs to rise faster than linearly with the flux; he used a *ϕ* ^2^ dependence for this in both model types. Such nonlinear self-enhancement is characteristic of self-organizing patterning mechanisms, for example interactions described by Turing (1952) reaction-diffusion theory for periodic concentration patterns.

**Figure 2:**
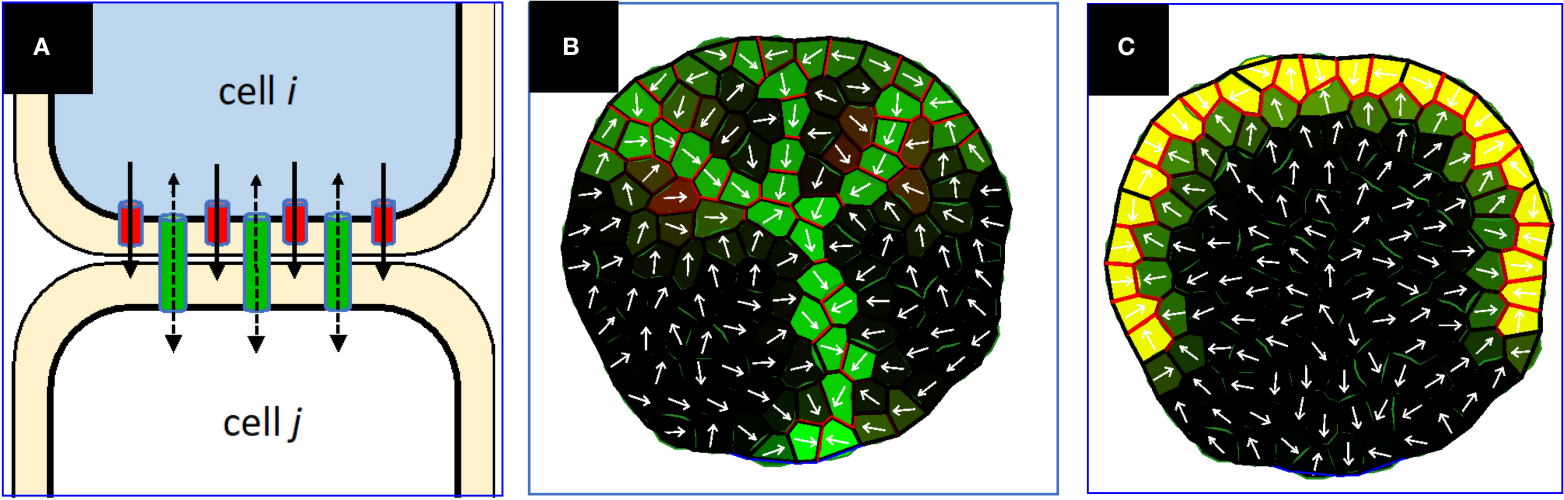
**(A)** Schematic of auxin flow (black arrows) via intercellular PD (green) and plasma membrane bound PIN (red). In this diagram, the auxin concentration difference (higher auxin concentration shown as blue) creates a diffusive flow through PD (dashed black arrows) from *i* to *j*, which (via the WTF mechanism) allocates more PIN in cell *i* towards cell *j* than for cell *j* towards cell *i*, amplifying the total *i* to *j* flow. In this case, the UTG mechanism could allocate some PIN in cell *j* towards *i*, but as long as the net *i,j* PIN is greater from *i* to *j*, the PIN-dependent PAT aligns with the concentration gradient (solid black arrows). The single-headed black arrows indicate the directional efflux via PIN; the double-headed arrows indicate the non-directional flow in the PD channels. Net auxin flux, *ϕ*, is the total auxin crossing the wall in a unit of time. **(B, C)** Prior PIN-only models have been unsuccessful in generating extending canalized veins under PIN-PAT inhibition, examples from Holloway and Wenzel (2021). **(B)** Simulation of normal conditions, PIN permeability parameter = 3. Green – intracellular auxin concentration; red – PIN concentration; yellow – blend of high intracellular auxin and PIN; white arrows, net intercellular auxin flux. **(C)** Very strong PIN-PAT inhibition (PIN permeability parameter = 0.1) stops vein extension, unlike observations such as **Figures 1E, F**.

Subsequent experimental work has indicated that auxin transport between cells is primarily through membrane transporters (**Figure 2A**, red) or intercellular plasmodesmata (PD) channels (**Figure 2A**, green) rather than by simple diffusion. Simple diffusion of extracellular protonated auxin may occur through the cell membrane into cells (but is likely a minor pathway compared to influx via the AUX1 transporter, Rutschow et al., 2014), but deprotonated auxin in the higher pH intracellular environment is not favored to passively diffuse through the membrane out of cells (Bennett et al., 2014 review; Rubery and Sheldrake, 1974; Raven, 1975; Kramer, 2009).

PIN family efflux transporters, especially PIN1 in *Arabidopsis*, have a strong impact on polar auxin transport (PAT) and auxin patterning (e.g. Okada et al., 1991; Gälweiler et al., 1998), including leaf vein patterning (e.g. Mattsson et al., 1999, Mattsson et al., 2003; O’Connor et al., 2014; Verna et al., 2015, Verna et al., 2019). A series of mathematical modelling projects have characterized the qualities of a with-the-flux (WTF) allocation of PIN for vein patterning, in which PIN is allocated to the membrane in proportion to intercellular auxin flux *ϕ* and can develop non-homogeneous polarized distributions around a cell perimeter, as well as unequal amounts on either side of a shared wall between cells (**Figure 2A**, red; Rolland-Lagan and Prusinkiewicz, 2005; Feugier et al., 2005; Stoma et al., 2008; Farcot and Yuan, 2013; Walker et al., 2013). These models are Mitchison (1981) Type 2 mechanisms, demonstrating the ability of flow-dependent transporter regulation to successfully canalize auxin distributions in a variety of scenarios, including leaf vein patterning (Rolland-Lagan and Prusinkiewicz, 2005; Feugier et al., 2005).

WTF models generally describe PIN1 polarization away from high concentration auxin sources, as seen for veins extending into the leaf (**Figure 1C**, red arrows; Scarpella et al., 2006; Wenzel et al., 2007; Bayer et al., 2009). However, PIN1 polarization in some cells is observed to be the opposite of this — i.e., towards high auxin sources (**Figure 1C**, white arrows) — for example at the convergence points (CPs, **Figure 1C**) in leaf margins from which veins extend. A series of up-the-gradient (UTG) models have been developed for such phenomena (Jönsson et al., 2006; Smith et al., 2006; Merks et al., 2007; Bilsborough et al., 2011). In UTG models, PIN is allocated to the membrane according to a neighbor cell’s auxin concentration, unlike the Mitchison Type 2 auxin-flux-dependent WTF models. The UTG mechanism can produce narrow tracks of auxin and PIN polarization (Merks et al., 2007), and could represent an additional means for canalization (supported by the observation of inwards PIN alignments along the lateral walls of veins, towards high in-vein auxin; purple arrows, **Figure 1C**; also see **Figure 2** of Bayer et al., 2009).

Combining both UTG and WTF PIN allocation mechanisms reproduces key features of auxin and PIN1 localization observed in leaf vein pattern development. These include: formation of primary and secondary vein initiation sites at marginal CPs; inward flow of auxin from CPs; canalization of PIN1 and auxin into narrow provascular tracks (Bayer et al., 2009; Cieslak et al., 2015; Hartmann et al., 2019); as well as the connection of secondary veins to primary veins (with high auxin levels), and the response of venation to pharmacological inhibition (such as with N-1-naphthylphthalamic acid, NPA) of auxin flow through PIN (Holloway and Wenzel, 2021). While WTF, UTG and UTG+WTF models quantify features of intercellular flows and provide mechanisms for the dynamic formation and alteration of provascular auxin and PIN patterns, the molecular details of either flux-sensing (WTF) or neighbor-cell concentration sensing (UTG) are not known. Proposed sensing mechanisms include tally molecules (Mitchison, 1980; Cieslak et al., 2015) and dynamics in the apoplast between cells (Wabnik et al., 2010). These models have focused on the initiation and canalization of provascular auxin tracks for the margin-originating primary and secondary veins (**Figure 1D**, red and blue). A model by Kneuper et al. (2021) considers factors likely to be important later, such as leaf-interior auxin synthesis (auxin synthesis mutations in *Arabidopsis* have strong effects on tertiary and above leaf-filling veins) and the effect of mechanical forces on cell elongation (which occurs after provascular track formation, as visualized with the auxin-responsive *ATHB* marker; Scarpella, 2024).

Despite the strong role of auxin flow through PIN, increasing evidence indicates significant non-PIN contributions to leaf vein patterning and canalization. Even when auxin transport through PIN is strongly inhibited (PIN-PAT-i), either by multiple *PIN* mutations (**Figure 1E**; Verna et al., 2019) or by treatment with drugs such as NPA (**Figure 1F**; Mattsson et al., 1999, Mattsson et al., 2003; Verna et al., 2019), leaf vein patterns are altered from normal WT (wild-type; **Figure 1D**) but not eliminated. (While it has been directly shown that NPA competes with auxin binding to PIN1 (Yang et al., 2022), the similar phenotypes in **Figures 1E, F** — much stronger than the phenotype of *pin1* alone (Verna et al., 2019) — suggest that NPA has effects across the PIN family.) Specifically, in conditions where auxin flow through PIN is largely or possibly entirely absent, veins are more numerous, tend to bundle (run in parallel), and do not connect or extend normally, but canalization is maintained, at both the auxin track (Mattsson et al., 2003) and differentiated vein (Mattsson et al., 1999; Verna et al., 2019) levels. There appears to be a limit to which altering auxin flow through PIN can affect leaf vein patterns: combining both multiple *PIN* mutations and strong NPA treatment produces patterns that appear similar to either treatment alone (**Figure 1G**; Verna et al., 2019).

Auxin flow through plasmodesmata (PD) intercellular channels likely constitutes at least part of the non-PIN aspect of leaf vein patterning and canalization: Linh and Scarpella (2022) showed that in strong PIN-PAT-i conditions (via NPA treatment) both WT leaves (**Figure 1I**) and narrowed-PD *cals3* mutants (**Figure 1J**) retained canalization and shared features such as more and bundled vein strands (with patterning differences along the margin); while wide-aperture-PD *gsl8* mutants (**Figure 1K**) in contrast lacked this canalization and exhibited broad regions of non-localized vein tissue. This was associated with changes in PD permeability during development: in WT and *cals3*, but not in *gsl8*, early relatively isotropic flows become increasingly polarized longitudinally within veins as compared to laterally from veins to surrounding tissue (Linh and Scarpella, 2022). These results indicate that in addition to the well-studied role of PIN in canalization, regulation of flow through PD can also canalize vein tissue. (Experimental evidence has not yet shown that other transporters — such as ABCB efflux proteins (e.g., Geisler et al., 2005) that co-regulate with PIN in the root (Mellor et al., 2022), or auxin influx carriers like AUX1/LAX family members (e.g., Swarup et al., 2001; Marchant et al., 2002) — play a role in leaf vein patterning and canalization when PIN activity is reduced, though recent modelling indicates that synergistic effects between ABCB and PIN help maintain apoplastic auxin gradients, Geisler and Dreyer, 2024).

Studies in other tissues also support roles for both PIN and PD regulation of auxin flows. In roots, the model of Grieneisen et al. (2007) showed the role of mature fixed PIN distributions (not dynamically self-organizing as during leaf vein canalization) in maintaining an auxin maximum at the root tip. A subsequent model with more complete transporter dynamics and regulation of auxin markers (Band et al., 2014) found discrepancies in model output and experimental data, which could be largely corrected by accounting for transport through PD (Mellor et al., 2020). PD and PIN also operate in parallel in the abaxial mid-rib epidermis of mature *Arabidopsis* leaves, where polarized auxin flows have greater longitudinal than lateral flow (Gao et al., 2020; Li et al., 2024).

Many factors can affect PD permeability between cells, such as the number and arrangement of PDs (e.g. in roots, Gunning, 1978; Zhu et al., 1998), the type of PDs (e.g. branched vs simple, Oparka et al., 1999), pressure (Park et al., 2019), the charge of solute molecules (Howell et al., 2024; which may favor anions such as cytoplasmic auxin), or callose deposition (affected in *cals3* and *gsl8*, Vaten et al., 2011). Some of these are auxin-dependent, such as the gating of PD by auxin-GSL8 feedback in hypocotyls (Han et al., 2014), or the interaction of auxin with the PDLP5 PD regulator in roots (Sager et al., 2020).

Linh and Scarpella (2022) reported that the rate of PD polarization and canalization during leaf vein development was also auxin-dependent, and that the results were consistent with a Sachs/Mitchison type canalization mechanism with feedback between auxin flow and PD permeability. In contrast to PIN transport, with unequal permeability in each direction across a wall (**Figure 2A**, red), permeability through intercellular PD pores would be expected to be equal in each direction through the wall (**Figure 2A**, green). Where PIN transport corresponds to the Mitchison (1981) Type 2 mechanism, PD transport corresponds to the Mitchison (1980) Type 1 mechanism. In general, Type 1 dynamics can create multicellular canalized tracks with source to sink directionality (Rolland-Lagan and Prusinkiewicz, 2005; Cieslak et al., 2021), but biological data points to this involving the regulation of symplastic PD to produce polarized PD permeability (Linh and Scarpella, 2022; and hypothesized by Mitchison, 1980) rather than involving facilitated diffusion in simple transmembrane diffusion (e.g. Bennett et al., 2014).

Experiments and modelling since Sachs’ (1978, 1981, 1991) original formulation of a canalization hypothesis indicate that there are at least several transport mechanisms contributing to canalization. The most studied involve auxin transport dynamics through PIN. Here, the two tendencies for PIN allocation, WTF and UTG, can each contribute to canalization in separate ways (WTF is a Mitchison Type 2 mechanism, UTG is not flux-dependent and is therefore not a Mitchison type mechanism). More recently, PD have been shown to be involved in auxin canalization, consistent with a Mitchison Type 1 mechanism. The mechanism for vein canalization has contributions from at least these three components: PIN-WTF, PIN-UTG, and PD. Initial computations of PIN-WTF and Mitchison Type 1 facilitated diffusion indicate the potential for patterning and canalization with combined PIN-PD mechanisms (Cieslak et al., 2021).

In this work we present a model with all three components. This combines a recent PIN-only model for primary and secondary leaf vein patterning and canalization (for which both UTG and WTF are indicated; Holloway and Wenzel, 2021; **Figure 2B**) with dynamic PD regulation. Fitting the combined model to data for PIN and PD perturbations provides insight into the relative contributions of the components to the overall canalization mechanism. The PD-flow component of the model is developed using data from strong PIN-PAT-i conditions. Flow through PD removes the unrealistic (for auxin) assumption of simple through-membrane diffusion contained in prior models. Addition of the PD flow enables the model to generate the extending, canalized veins observed when PIN transport is strongly reduced or removed, as well as generating the dynamic increase in PD canalization during vein development and the dependence of canalization on PD aperture. The addition of PD dynamics clarifies the canalization hypothesis in terms of current data and current mechanistic proposals for PIN and PD regulation of intercellular vein patterning and canalization in leaves. This supports research into other components of leaf vein development, such as the role of other transporters and of auxin signaling dynamics.

## 2 Model and methods

In a set of connected cells representing a leaf (e.g. **Figures 2B, C**), the mathematical model describes the rates of change of:

the intracellular auxin concentration in each cell (*A_i_*)


(1) $$\frac{dA_{i}}{dt}=auxpr\cdot A_{\text{prec},i}-auxdec\cdot A_{i}+T\sum_{j}\left(\frac{P_{ji}A_{j}}{1+A_{j}}-\frac{P_{ij}A_{i}}{1+A_{i}}\right)+D\sum_{j}D_{ij}(A_{j}-A_{i});$$

the PD between cell *i* and neighbor cell *j* (*D_ij_*; **Figure 2A**, green)


(2) $$\frac{dD_{ij}}{dt}=\alpha \phi^{2}+\beta -\gamma D_{ij};$$

the PIN concentration in each cell (*P_i_*; where it is synthesized and degraded)


(3) dPidt=pinpr·Ai−pindec·Pi−∑jdPijdt;

and the transmembrane PIN in cell *i* towards cell *j* (*P_ij_*; **Figure 2A**, red), allocated from the cytoplasm


(4) $$\frac{dP_{ij}}{dt}=\frac{k_{U}P_{i}f(A_{j})}{1+P_{i}}+\frac{P_{i}}{1+P_{i}}\left(k_{Wq}\phi^{2}+k_{Wl}\phi \right)-k_{\text{off}}P_{ij}$$.

The PIN-auxin dynamics in Equations 1, 3 and 4 were developed in Holloway and Wenzel (2021) from prior WTF and UTG models (Rolland-Lagan and Prusinkiewicz, 2005; Merks et al., 2007) and used to model primary and secondary vein patterning in normal and NPA-treated conditions. To this is added auxin flow through PD (Equation 1, last term) and PD regulation (Equation 2; from Mitchison, 1980). The dynamics in Equations 1–4 generate canalized tracks of high auxin concentration across the cellular representation of the leaf.

In Equation 1, the change of auxin in each cell *i*, the first two terms are for auxin production (*auxpr*, from precursor *A*_prec_) and decay (*auxdec*). Production occurs in zones along the leaf margin where *A*_prec_ > 0 (Holloway and Wenzel, 2021), with successive activation of the primary vein (Zone 1, Z1, *A*_prec_ = 0.5 at *t* = 0, then increasing by 0.0001/s) and secondary veins (Zone 2, Z2, *A*_prec_ = 0 at *t*< 2 h 45 m, then increasing by 0.0001/s). (See Introduction regarding a marginal source for primary and secondary veins; production is modelled in the outermost cells, but could correspond to several marginal layers in the leaf.) The third and fourth terms in Equation 1 are for auxin transport between cell *i* and its neighbors *j*: the third term is for transport through PIN, multiplying PIN permeability *T* by the number of PIN transporters (*P_ij_* for PIN in *i*’s plasma membrane, **Figure 2A** red; *P_ji_* for PIN in *j*’s plasma membrane; Holloway and Wenzel, 2021; Merks et al., 2007); the fourth term is for transport through PD, multiplying permeability *D* by the PD between cells *i* and *j*, *D_ij_* (with *D_ij_* = *D_ji_* because PD are symplastic pores between cells). Cells are assumed to be well-mixed (Merks et al., 2007), with transport rate limited by intercellular exchange (Rutschow et al., 2011).

Equation 2 describes the change in PD. *D_ij_* represents the total PD cross-sectional area between cells, which could correspond to the number of PDs or to the cross-sectional area (aperture) of individual PDs. The β and γ terms represent background, auxin-flow independent production and decay of total PD area, respectively. The α term is the Mitchison (1980) formulation for the feedback between PD and intercellular auxin flux *ϕ* to produce canalization. Prior PIN-only models used simple cross-membrane diffusion for the 4^th^ term in Equation 1: as well as being unrealistic for auxin transport (see Introduction), this assumed that diffusion occurs freely through all positions along the interface between two cells — rather than depending on the number and size of the intercellular channels — resulting in artefacts in vein paths due to artificially favoring flow through long walls compared to short walls.

Parameter selection was sequential, first for the PD-only component of the model (*T* = 0 in Equation 1; plus Equation 2), corresponding to strong PIN-PAT-i conditions, then later adding PIN dynamics (*T* > 0 in Equations 1, 3, 4). Parameters were found for the PD-only component (**Table 2**) that generate extending, canalizing strands with the following considerations:

- Time: in computational hours, minutes and seconds. These units are estimated to scale by a factor of 10 to biological values in absolute terms: for normal conditions, primary and secondary vein patterns develop in 7h in computational time, a stage reached by 3 days post-germination in *Arabidopsis* first rosette leaves (Mattsson et al., 2003). Relative time to develop in simulations reflects biological results qualitatively, for example with patterns developing more slowly in PIN-PAT-i than normal conditions.
- Auxin production (*auxpr*, *A*_prec_, *auxdec*): values from Holloway and Wenzel (2021) produce sustained auxin sources and cross-leaf auxin traces with high auxin concentration. Extension of a concentration gradient from a source depends on source strength (*auxpr*, *A*_prec_), decay (*auxdec*) and transport (e.g. Harrison, 1993; Holloway et al., 2023). Lower *auxpr*, *A*_prec_ or increased *auxdec* decrease vein extension and in-vein auxin concentration. For transport, lower permeability *D* shortens auxin expansion from the source and high *D* floods the leaf with auxin. At *D* = 0.8 (**Table 2**), strands extend halfway across the leaf by *t* = 15 hours with PD-only (PIN-PAT-i; Equations 1, 2 with *T* = 0) and fully across the leaf in 7h for normal PIN + PD conditions (Equations 1–4; *T* = 6).
- The β/γ ratio (PD increase / PD decrease) gives the steady-state value of *D_ij_*, i.e. the background total between-cell PD area apart from the influence of auxin flow on PD. β/γ = 0.6 (**Table 2**) allows canalization and extension. Lower than this shortens extension, higher than this begins to overwhelm canalization and lead to auxin leakage through the leaf. The α term (auxin flux dependent PD cross-sectional area increase) produces canalization: lower α reduces canalization; higher α increases canalization. α = 0.0001 produces strands with PD-only (*T* = 0 in Equation 1; PIN-PAT-i conditions). Higher α than this interferes with PIN canalization when *T* > 0 (PIN + PD transport).
- The total permeability across a wall (*D* x *D_ij_*; 0.8 x 0.6 = 0.48 cell/s, **Table 2** values) at early stages before significant flow-induced canalization corresponds to 4 µm/s, using typical cell lengths of 9 µm in leaves during primary and secondary vein development (Wenzel et al., 2007). This is in the range of measured PD permeabilities: in mature roots, 8.5 µm/s for carboxyfluorescein (Rutschow et al., 2011); and for fluorescein in mature leaves and stems, from approx. 0.6 – 2 µm/s for isotropic flow in the stem and pavement cells, to 3.5 – 5 µm/s for anisotropic longitudinal flow in the midrib and petiole (Gao et al., 2020). Modelling of flow through individual PD converges to these measured values for overall intercellular permeability (Deinum et al., 2019). The 4 µm/s value used in our model converts to an effective diffusivity via PD of 26 µm^2^/s for the intercellular scale, including both cytoplasmic intracellular transport and transport between cells (following Rutschow et al., 2011). Absolute biological values may be slower. For example, with the residual non-PIN transport in strong PIN-PAT-i conditions veins extend approximately 500 µm between 5 and 10 days post-germination (Mattsson et al., 1999). Simply considering diffusion, this corresponds to an effective diffusivity of 0.3 µm^2^/s (using $D=x^{2}/2t$ ; consideration of auxin decay and source strength could raise the estimated diffusivity). Timescales (**a**) and lower cell number resolution may contribute to faster rates in the model than in the leaf in absolute terms. For this reason, we focus on predictions regarding relative developmental timing, such as the slow down of strand extension in PIN-PAT-i conditions from normal.

**Table 2:** Model components, PD terms (Equations 1, 2).

Component	Definition	Value^a^
cells *i* and *j*	Adjacent (neighbouring) cells	
*t*	Computational time	
*A*_i_ *, A*_j_	[Auxin] in cell *i* or *j*	
*A*_prec,_ *_i_*	Auxin precursor in *i*	
*auxpr*	Auxin production rate constant	30 ^b^
*auxdec*	Auxin decay rate constant	0.5 ^b^
*D*	PD permeability constant	0.8
*D_ij_*	Total PD cross-sectional area in *ij* wall	
*ϕ*	Intercellular auxin flux	
α	Rate constant, *ϕ*-dependent *D_ij_* increase	1e-4
β	Rate constant, *ϕ*-independent *D_ij_* increase	3e-2
γ	Rate constant, *ϕ*-independent *D_ij_* decay	5e-2

a in computational units for time (s), concentration (amount/cell) and distance (cell).

b values from Holloway and Wenzel (2021).

PIN dynamics are represented by Equation 3 (with intracellular production, *pinpr*, and decay, *pindec*) and Equation 4 for the allocation of PIN to the plasma membrane (of cell *i* towards cell *j*, *P_ij_*). In Equation 4, $f(A_{j})=100A_{j}/(100+A_{j})$ . Equation 4 represents the two polarization mechanisms indicated for PIN: UTG allocation is regulated by the first (*k_U_*) term; WTF allocation is regulated by the 2^nd^ term (*k_W_* terms; with the intercellular auxin flux *ϕ* dependence corresponding to Mitchison (1981) Type 2 mechanism for transporter-dependent flow (via *P_ij_*); see also Rolland-Lagan and Prusinkiewicz, 2005).

The total auxin flux *ϕ* is computed from the sum of the *T* and *D* terms in Equation 1 at the start of each time step. Combining the flux from both PIN and PD is supported by results from Linh and Scarpella (2022): 1) PIN-PAT-i delays the normal reduction of PD permeability involved in canalization; and 2) PD aperture mutations affect the normal continuity of PIN1 expression in provascular tracks.

Parameter values for Equations 3, 4 (see **Table 3**) were developed in Holloway and Wenzel (2021) for PIN-dependent generation of primary and secondary vein patterns. With PD flow, WTF does not need to be as strong: *k*_Wl_ and *k*_Wq_ are lower than in the PIN-only model (Holloway and Wenzel, 2021). The PIN permeability constant (*T* = 6) is higher than the PD permeability constant (*D* = 0.8) in part due to the saturation term (denominator) in the PAT term in Equation 1 (from Merks et al., 2007). Raising *T* (> 6) showed little effect in simulations, while lowering *T* had effects consistent with NPA treatment (see Results). Biologically, the permeability of PIN and PD may be of similar magnitudes, with estimates of 1 – 5 µm/s through PIN from auxin velocities in mature vasculature (Kramer et al., 2011). However, due to the changing distributions and polarization of PIN and PD during vein formation and canalization, overall wall permeabilities are highly dynamic. Equations 2 and 4 model the relative PIN and PD contributions to wall permeability as the *P_ij_* and *D_ij_* undergo such changes. Parameters in **Tables 2** and **3** represent normal vein patterning. Variation in particular parameters (see Results) produces features of mutant and PIN-PAT-i patterns. Relative constraints on canalization parameters are summarized in **Table 4**.

**Table 3:** Model components, PIN terms (Equations 3, 4).

Component	Definition	Value^a^
*P*_i_	Cytoplasmic [PIN] in cell *i*	
*P*_ij_	PIN in membrane of cell *i* towards cell *j*	
UTG	Up the gradient PIN allocation	
WTF	With the flux PIN allocation	
*T*	PIN permeability constant	6
*k*_U_	UTG allocation rate constant	4e-3^b^
*k*_Wl_	Linear WTF allocation rate constant	3e-3
*k*_Wq_	Quadratic WTF allocation rate constant	1e-5
*k*_off_	PIN membrane detachment rate constant	4e-3^b^
*pindec*	PIN decay constant	1e-2^b^
*pinpr*	PIN production constant	5e-3^b^
*C*	Margin-interior PIN efficiency	0.833^b^

a in computational units for time (s), concentration (amount/cell) and distance (cell).

b values from Holloway and Wenzel (2021).

**Table 4:** Relative constraints on canalization.

Parameter	Too low	Too high
*k_Wl_*	reduced vein extension from source	poor CP formation and canalization
*k_Wq_* and α	broad uncanalized auxin distributions	poor source to sink directionality

All simulations were run in three different leaf tissue representations to demonstrate the robustness of the vein patterning results to variability in cell regularity and tissue geometry:

- Fixed-size, non-growing tissues with irregular cell size and shape (shown in the main text Figures).
- Tissues undergoing growth and cell division (irregular cell size and shape). Growth algorithm as in Holloway and Wenzel (2021). Growth rates, see **Table 5**. Division occurs when cells double in size. These results are shown in the **Supplementary Material**.
- Fixed-size (non-growing) tissues with equal cell size and shape (square). Results are shown in **Supplementary Material**.

**Table 5:** Growth rates.

Parameter	Definition	Value
*G*_1_	Cell growth in auxin producing cells	0.2
*G*_2_	Cell growth in Z2 while *A*_prec_ ≤ 0	1.4
*G*_3_	Cell growth in all other cells	0.6

The model was implemented in the cell-based software package VirtualLeaf, version 1.0.3 (Merks et al., 2011; Merks and Guravage, 2013; Antonovici et al., 2022; https://code.google.com/archive/p/virtualleaf/, compiled in Windows with Qt Creator 11.0.3). The model specification (PINPD.cpp) and a leaf geometry and parameters file (Fig4a.xml) can be downloaded from https://github.com/davidhollowaybc/PINPD.

## 3 Results

### 3.1 Auxin canalization via plasmodesmata, strong PIN-PAT-i conditions

Experiments curtailing auxin transport through PIN, either by *PIN* mutation (Mattsson et al., 1999; Verna et al., 2019) or via drugs such as NPA interfering with PIN-dependent PAT (Mattsson et al., 1999, Mattsson et al., 2003; Linh and Scarpella, 2022), indicate a residual patterning system that forms canalized strands of auxin that subsequently vascularize. Mutations affecting PD aperture size suggest that this residual system involves dynamic control of the PD area between cells (Linh and Scarpella, 2022).

**Figure 3:**
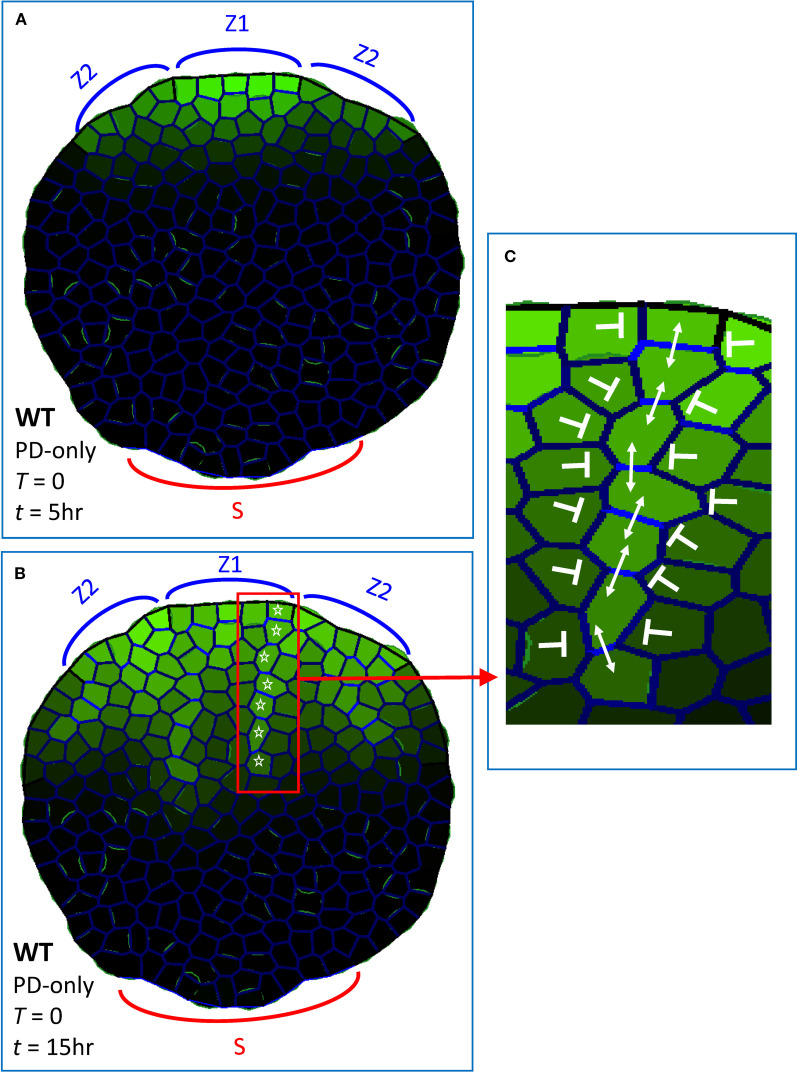
Dynamic plasmodesmata (PD) regulation produces extending provascular strands of high auxin, as observed experimentally when PIN-dependent PAT is absent or severely curtailed, in multiple *pin* mutants or with strong NPA doses. These simulations (using Equations 1, 2) had no flow through PIN (*T* = 0, Equation 1), auxin transport was only through PD (i.e, PD-only model). Green intensity in cells, intracellular auxin concentration (*A_i_*; Equation 1); blue intensity on walls, total PD area between cells (*D_ij_*, Equations 1, 2). Auxin was produced in margin zones, with Z1 initiating (A_prec_ > 0; Equation 1) at *t* = 0 and Z2 initiating at *t* = 2 hr 45 min, corresponding to the sequential initiation of primary and secondary veins in normal development (**Figure 1D**, red and blue respectively). Sink cells (S) at the base of the leaf had strong auxin decay and represented the vasculature of the rest of the plant. Auxin patterning in a non-growing leaf shape with irregular cell size and shape, at **(A)** *t* = 5 hr and **(B)** *t* = 15 hr, showed increasing canalization and extension in time. White stars indicate one of the provascular strands formed. **(C)** enlargement from **(B)**, showing high PD within the strand (double-headed arrows) and low PD between the strand and neighbouring cells (blunt arrows). (Bright green on walls seen on some proximal cells is due to the graphical representation of cell outlines, and is not related to auxin content.)

These strong PIN-PAT-i conditions were simulated by the PD-only model (Equation 1, 2; with PIN permeability *T* = 0); i.e. with PIN-dependent PAT off and all auxin transport through the *D* term in Equation 1. As shown in **Figure 3**, these PD-only dynamics created multiple auxin strands flowing from margin source regions (Z1, Z2) into the leaf, corresponding to experimental observations (Mattsson et al., 1999; Verna et al., 2019). Previous models, which combined PIN-dependent PAT with simple Fickian diffusion (e.g. Rolland-Lagan and Prusinkiewicz, 2005; Merks et al., 2007; Bayer et al., 2009; Holloway and Wenzel, 2021) could not do this at *T* = 0: lacking a non-PIN canalizing mechanism, auxin did not form provascular strands and became restricted to the margin source as PIN permeability (*T*) approached zero (e.g., **Figure 2C**; compare to **Figure 2B**). Experimental observations of stranding in strong PIN-PAT-i conditions suggest the presence of a non-PIN canalizing mechanism: **Figure 3** demonstrates that this mechanism could involve feedback between auxin flow and wall permeability through PD (Equation 2; Mitchison, 1980 Type 1 mechanism).

Experiments indicate that in strong PIN-PAT-i conditions, extension of auxin strands is delayed compared to normal development: constrained near the margin source in early stages, but extending as provascular strands later (Mattsson et al., 1999, Mattsson et al., 2003). Simulations of PD-only patterning showed a similar progression (**Figures 3A** to **B**). Provascular strands also became increasingly narrow and canalized in time, with increasing contrast between high PD flow in the direction of the strand and low PD flow between a strand and its surroundings (**Figure 3C**). This corresponds qualitatively to the Linh and Scarpella (2022) observations of a transition from un-canalized to canalized PD flow over approximately 2 days of development. The PD-only canalization and formation of provascular strands is robust to variation in cell arrangement, cell growth and cell division: **Supplementary Figure S1B’** shows stranding that developed during growth and cell division from a smaller size (**Supplementary Figure S1B**); **Supplementary Figures S1C, C’** show stranding on a grid of regular, square cells of equal size (the square geometries being less leaf-like, stranding was tested from one production zone, Z1, rather than modelling the multiple sources associated with a vein network).

### 3.2 PIN + PD dynamics, normal venation and intermediate PIN-PAT-i

**Figure 4:**
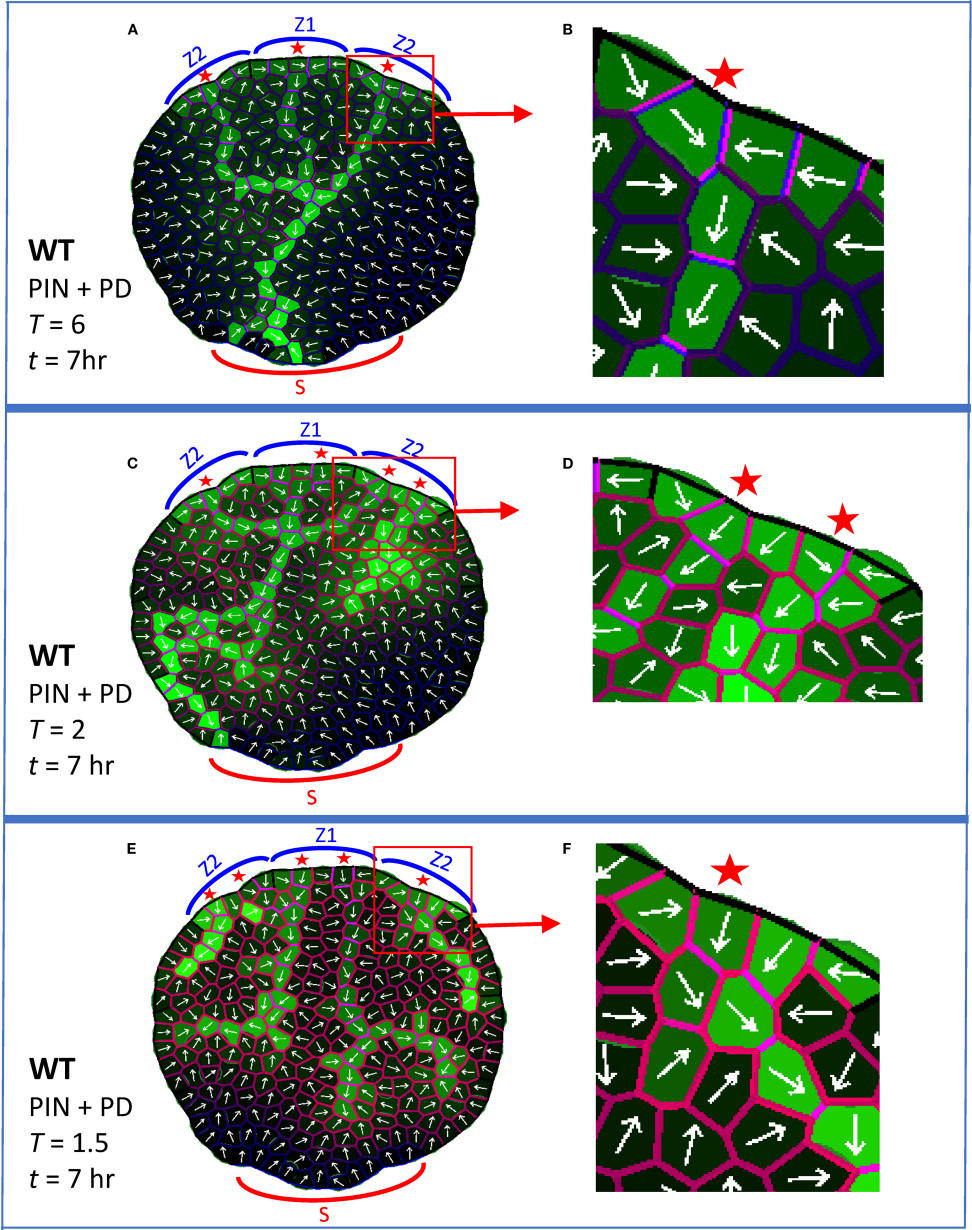
PIN + PD dynamics form normal provascular auxin patterns and also respond to a graded decrease in PIN-dependent PAT (e.g. by NPA treatment). In addition to previous colors (**Figure 3**), red cell wall intensity indicates membrane-bound PIN (*P_ij_*, Equations 1, 4); the violet mix indicates high PIN (red) and high PD (blue). White arrows indicate net PIN-dependent auxin flux for each cell. Auxin is produced in the Z zones, as in **Figure 3**. All simulations shown at *t* = 7 hr. **(A, B)** At normal auxin permeability through PIN (parameter *T* = 6; Equation 1), a CP formed in each Z zone in the margin (red stars) and initiated a provascular auxin stream into the leaf. The central primary vein connected to the basal sink S and secondary veins attached to the primary. **(B)** Enlargement showing the PIN and PD distributions associated with a CP and canalized vein. **(C, D)** At somewhat reduced *T* (*T* = 2), corresponding to moderate NPA treatment, patterning defects began to appear, including increased numbers of CPs, poorer canalization (shown enlarged in D) and poorer source-sink directionality. **(E, F)** At more strongly reduced *T* (*T* = 1.5), corresponding to stronger NPA treatment, the midvein split, dividing transport in the leaf into left and right halves, veins branched and vein extension was further reduced (CP region enlarged in **(F)**).

In normal conditions (WT, no NPA) auxin is transported between cells by both PIN and PD. Modelling these dynamics (Equations 1–4, *T* = 6) produced single canalized provascular auxin traces from each primary (Z1) and secondary (Z2) source zone (**Figure 4A**). PIN dynamics in each source zone produced the ‘reverse fountain’ PIN alignment (**Figure 1C**) with one CP (**Figure 4** red asterisks) from which the provascular trace extended into the leaf. Without dynamic PIN, PD-only dynamics formed multiple canalizing provascular strands from each source zone (**Figure 3B**), corresponding to observations in strong PIN-PAT-i conditions. This normal CP-to-extension patterning also robustly formed during growth and cell division (**Supplementary Figure S2B**), and on a regular, square grid (**Supplementary Figure S2C**).

Vein extension is faster in normal conditions than in PIN-PAT-i conditions. Experimentally, primary veins in *Arabidopsis* reach the leaf base by 5 days in normal conditions; in moderately NPA-treated leaves, veins have not left the distal leaf margin source in that time, but do extend over halfway across the leaf by 10 days (Mattsson et al., 1999). Similarly, in simulations of normal development (PIN + PD) the primary vein crossed the leaf and touched the basal sink by *t* = 2hr 30min (earlier than the stage shown in **Figure 4A**), while the PD-only strands in **Figure 3B** crossed only half the leaf by *t* = 15hr. These modelling results indicate that the observed speed-up of vein extension from PIN-PAT-i to normal conditions could be due to the additional auxin transport mechanism (PIN-dependent PAT) in normal conditions.

The full PIN + PD model also increasingly canalized auxin in time. This has contributions from PD (indicated by the provascular strands in strong PIN-PAT-i conditions and PD mutants; Linh and Scarpella, 2022), represented by the α term (auxin flux dependent increase in PD area) in Equation 2; and from PIN (indicated by PIN1 polarization in provascular traces; Scarpella et al., 2006; Wenzel et al., 2007; Bayer et al., 2009; Verna et al., 2019; Linh and Scarpella, 2022), represented by the *k_Wq_* (WTF) and *k_U_* (UTG) terms in Equation 4. In the full PIN + PD model, PD-canalization (see blue walls, **Figure 3C**) and PIN-canalization (see red walls, **Figure 2B**) co-localize (violet merge, **Figures 4AB**): polarized PIN efflux combines with the anisotropic PD distribution (biased in the direction of the vein) to produce high flow along but not between veins.

Increasing NPA dosages produce increasingly strong defects in normal venation patterns (see for example Mattsson et al., 1999, Mattsson et al., 2003; Scarpella et al., 2006; Wenzel et al., 2007). While not as extreme as strong PIN-PAT-i effects, intermediate NPA treatments (or weak *PIN* mutations) induce extra CPs, inhibit the joining of secondary to primary veins, and slow vein extension and canalization compared to normal. The graded increase of PIN-PAT-i was simulated by decreasing *T* (Equation 1), the permeability of auxin through PIN. With moderately decreased *T*, the model produced extra CPs and poorly-canalized (broadened) veins (**Figure 4C**), corresponding to observations under moderate NPA treatment (Mattsson et al., 1999, Mattsson et al., 2003; Scarpella et al., 2006). More strongly decreased *T* led to vein splitting and further reduction of extension (**Figure 4E**); this included division of the leaf into left and right halves by splitting of the primary midvein, as observed experimentally (e.g. Mattsson et al., 1999, Mattsson et al., 2003; Verna et al., 2019). (Reduction of *T* to zero corresponds to strong PIN-PAT-i, or PD-only patterning, **Figure 3**.) At low *T*, the PIN distribution became more uniform and less polarized (**Figures 4D, F**) than at normal *T* levels (**Figure 4B**). The underlying PD dynamics maintained vein extension and canalization continuously as *T* (PIN-dependent PAT) decreased, unlike prior models, in which PIN provided the only canalizing mechanism and vein extension was lost at low PIN-dependent PAT (**Figure 2C**; Holloway and Wenzel, 2021; Bayer et al., 2009). The intermediate PIN-PAT-i effects were robust to patterning on a growing and dividing leaf (**Supplementary Figures S2B, E, H**) and on a regular square grid (**Supplementary Figures S2C, F, I**), showing one or more of the following: extra CPs, decreased canalization, or decreased vein extension.

### 3.3 PD-aperture mutants

Through *cals3* and *gsl8* mutants, Linh and Scarpella (2022) showed that changes in PD aperture affect vein patterning. Veins canalize in *cals3* smaller-than-WT PD aperture mutants, while canalization is reduced in *gsl8* larger-than-WT PD aperture mutants. The effects were stronger in *gsl8* with NPA treatment than without: in normal conditions, *gsl8* showed broadened veins and leakage of marker dyes; in PIN-PAT-i conditions, *gsl8* showed a loss of distinct veins.

Simulating PD-aperture mutations by altering the background level (β/γ ratio of auxin flux independent increase/decay in PD, Equation 2) of between-cell PD area (*D_ij_*) produced similar trends. With β/γ = 0.6 set for WT, *cals3* was represented by β/γ = 0.3 (halving the PD area) and *gsl8* was represented by β/γ = 1.2 (doubling the PD area).

**Figure 5:**
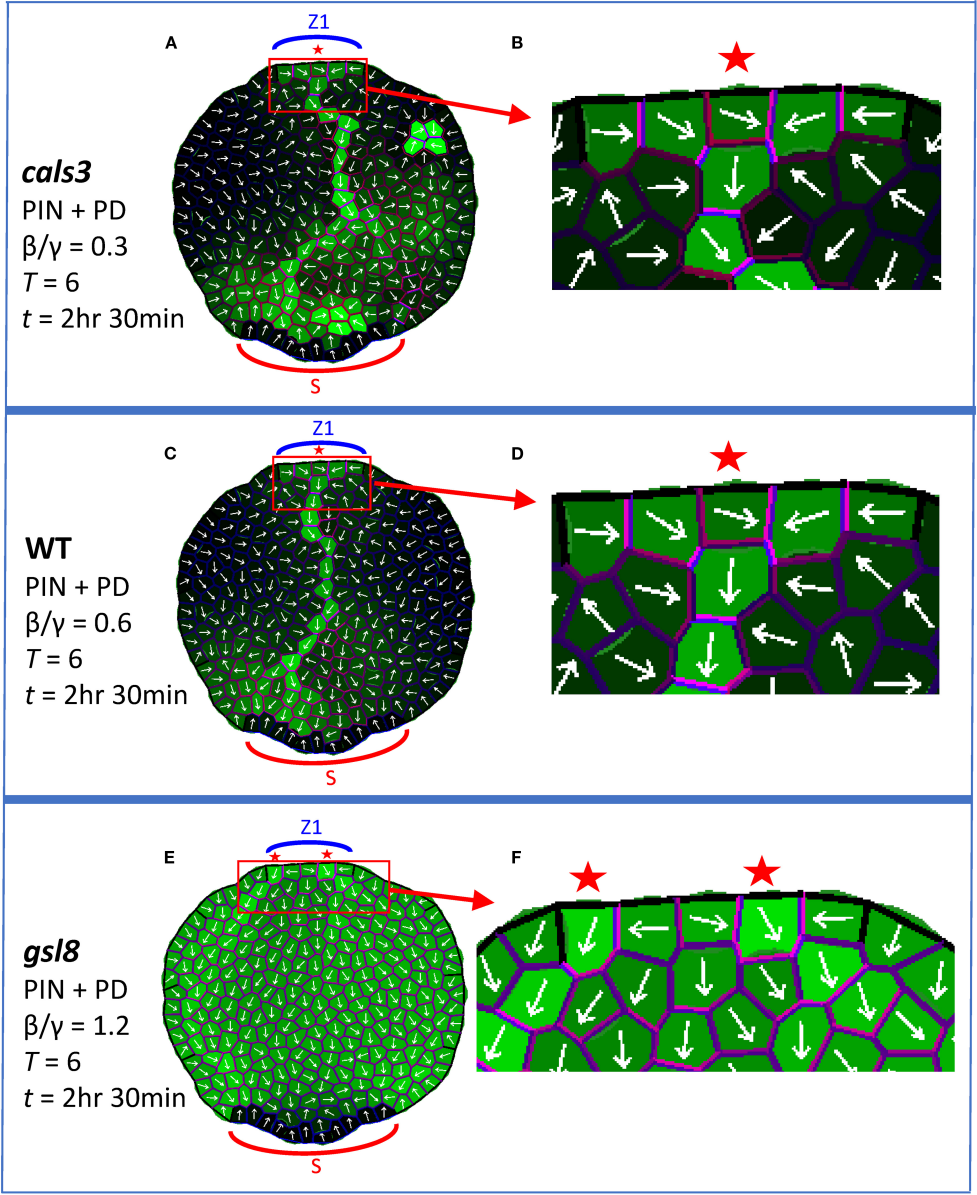
Auxin patterning responds to alterations of between-cell PD area. The full PIN + PD model is used, corresponding to WT, *cals3* and *gsl8* mutations without NPA treatment. Coloring and labelling as in **Figure 4**. All results are shown at *T* = 6 and *t* = 2 hr 30 min. Auxin is produced in the single indicated zone (Z1, the primary initiation zone) from *t* = 0. **(A)** *cals3* simulation (β/γ = 0.3, between-cell PD area half of WT) forms a canalized auxin strand; the CP area is enlarged in **(B)** to show transporter and channel anisotropy (violet). **(C)** WT simulation, with normal PD area (β/γ = 0.6), forms a canalized auxin strand; the CP area is enlarged in **(D)**. **(E)** *gsl8* simulation (β/γ = 1.2, between-cell PD area twice that of WT) shows weaker canalization than WT and auxin leakage. Enlargement in **(F)** shows the associated loss of transporter and channel anisotropy (compared to **(D)**).

With normal conditions (full PIN + PD model, Equations 1–4), these β/γ shifts produced canalization in *cals3* (**Figure 5A**) and WT (**Figure 5C**), and showed reduced canalization with auxin leakage into the leaf for *gsl8* (**Figure 5E**; the CP also split in this case). This is associated with a reduction in PIN transporter and PD channel distribution anisotropy (i.e. increasing uniformity) from *cals3* and WT (**Figures 5B, D**; violet) to *gsl8* (**Figure 5F**, violet). The diminished or lost canalization at higher β/γ also occurred with growth and cell division (**Supplementary Figures S3B, E, H**) and on a regular square grid (**Supplementary Figures S3C, F, I**).

**Figure 6:**
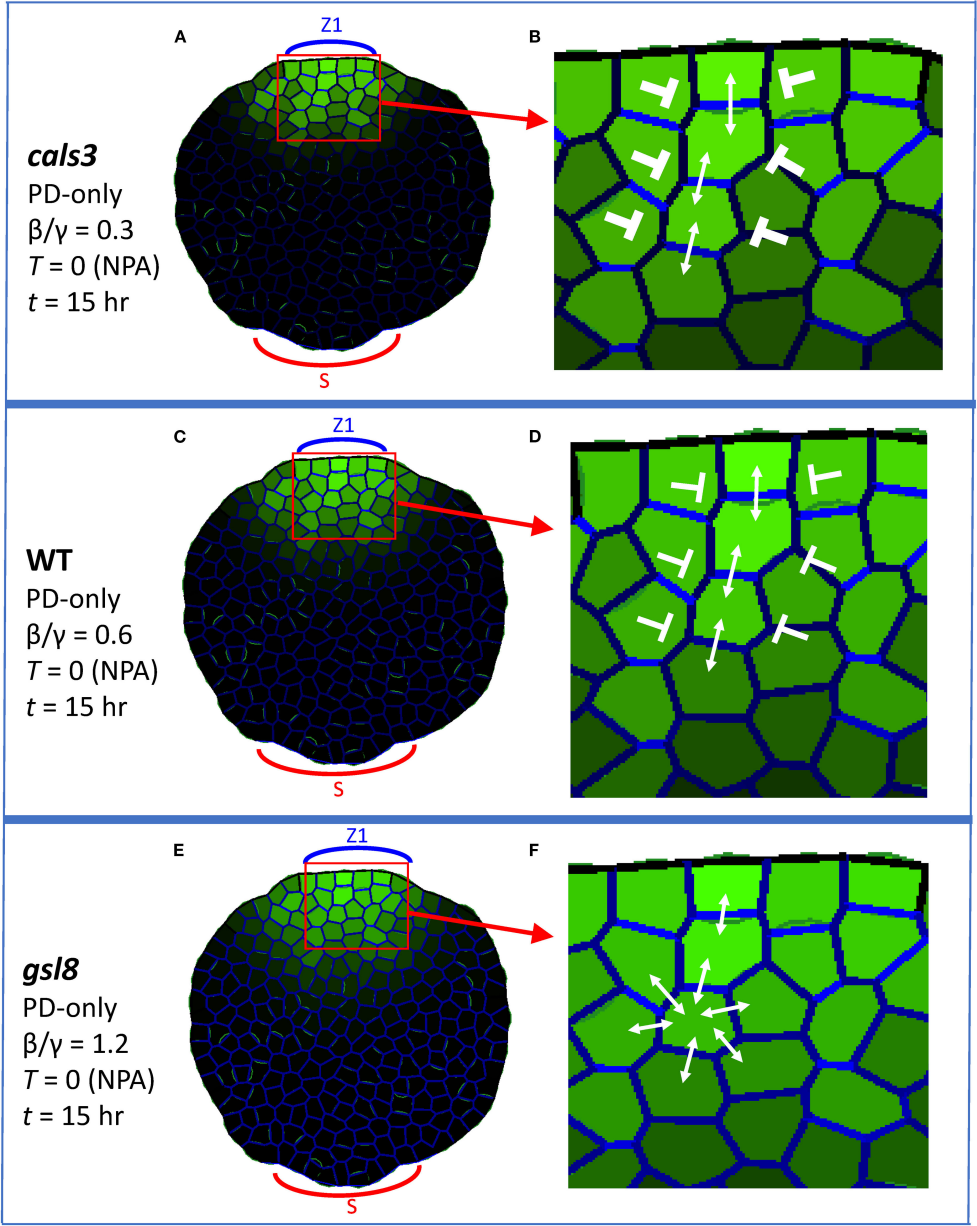
Auxin patterning in PIN-PAT-i conditions responds to alterations of between-cell PD area; PD-only model, corresponding to WT and *cals3* and *gsl8* mutants under strong NPA treatment (*T* = 0). Coloring and labelling are as in **Figure 3**. All results are shown at *t* = 15hr. Auxin is produced in the single indicated zone (Z1, the primary initiation zone). **(A)** *cals3* simulation (β/γ = 0.3, between-cell PD area half that of WT) forms auxin strands; enlarged in **(B)** to show PD alignment (weight of blunt arrows indicates inhibition strength for between-strand auxin flow). **(C)** WT strand formation at β/γ = 0.6, enlarged in **(D)** to show PD alignment (weaker inhibition of lateral auxin flow than **(B)**. **(E)** *gsl8* simulation (β/γ = 1.2, between-cell PD area twice that of WT) shows weakened strand formation, enlarged in **(F)** to show the more uniform PD distributions and auxin flow.

For strong PIN-PAT-i conditions, the PD-only model (Equations 1, 2; *T* = 0) produced distinct auxin strands from the source for *cals3* (**Figure 6A**) and WT (**Figure 6C**), in which PD were aligned within (not between) the strands (enlargements in **Figures 6B, D**). For *gsl8* (**Figure 6E**), PD distributions became more uniform, and were not aligned in strands (**Figure 6F**). In this case, the non-auxin flux dependent PD terms (β/γ) overcome the auxin-flux-dependent (α) term, reducing or eliminating canalization. Lost or diminished canalization for *gsl8* was observed with cell growth and division (**Supplementary Figures S4B, E, H**) and on the regular square grid (**Supplementary Figures S4C, F, I**).

### 3.4 Exogenous auxin

**Figure 7:**
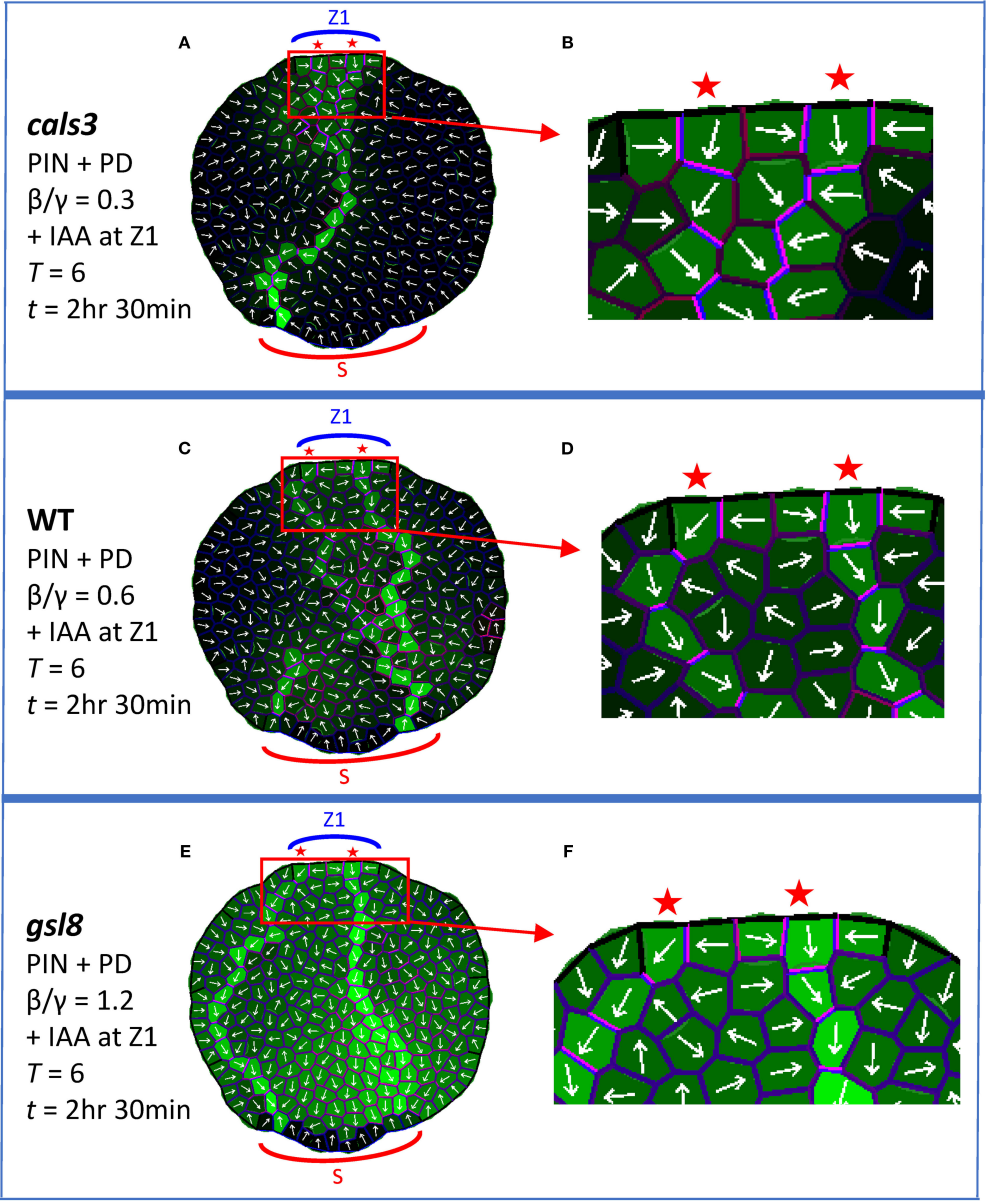
Localized boost of auxin production, corresponding to local application of IAA at Z1, induces extra veins. PIN + PD model used, with coloring and labelling as in **Figure 4**. All results are shown at *T* = 6 and *t* = 2 hr 30 min. Auxin is produced in the margin zone, Z1, with an initial precursor level of 2 (*A*_prec_ quadruple the normal value in **Figure 3**– **Figure 6**). **(A)** *cals3* simulation, PD area half of WT (β/γ = 0.3), enlarged in **(B)** to show the two CPs and anisotropic channel and transporter distributions (violet). **(C)** WT simulation (β/γ = 0.6), enlarged in **(D)** to show extra CPs and channel and transporter anisotropy. **(E)** *gsl8* simulation, PD area twice that of WT (β/γ = 1.2), enlarged in **(F)** to show the two CPs and less canalized auxin distribution.

Exogenously applied auxin (IAA) induces veins in WT (Verna et al., 2019) and to a lesser extent in *cals3* and *gsl8* (Linh and Scarpella, 2022); *gsl8* also shows vein broadening (Linh and Scarpella, 2022). Simulating exogenous IAA by an increase in source auxin precursor levels (initial *A*_pre_ *_c_* = 2; quadruple the levels shown in **Figures 3**–**6**) also produced extra veins: the normal single CP per source zone split to form two CPs, extending two veins. Two CPs were observed for boosted IAA with *cals3* reduced PD area (**Figure 7A, B**; β/γ = 0.3) and with WT PD area (**Figure 7C, D**; β/γ = 0.6), with retention of canalization; two CPs formed and auxin was less canalized (broader) with *gsl8* increased PD area (**Figure 7E, F**; β/γ = 1.2), corresponding to Linh and Scarpella (2022) observations. These results of CP splitting and *gsl8* loss of canalization were robust to cell growth and division (**Supplementary Figures S5B, E, H**) and on regular square grids (**Supplementary Figures S5C, I**),

## 4 Discussion

### 4.1 The need to model both PIN and PD in leaf vein patterning and canalization

Similar to results in mature leaf hyponasty (Li et al., 2024; Gao et al., 2020), hypocotyls (Han et al., 2014) and roots (Sager et al., 2020), recent evidence in leaf vein development also appears to involve auxin transport through both PIN and PD (Linh and Scarpella, 2022). A feature of leaf vein development is the canalization of early broad auxin concentration distributions to narrow provascular strands. The means by which this occurs, or the canalization hypothesis, has been discussed phenomenologically since Sachs (1978). Mathematical models of canalization have been developed in general (Mitchison, 1980; Mitchison, 1981) and with respect to PIN transport (e.g. Rolland-Lagan and Prusinkiewicz, 2005; Feugier et al., 2005; Bayer et al., 2009; Holloway and Wenzel, 2021), testing the dynamic features of auxin flow regulation that could produce canalization, consistent with experimental data on vein patterns, auxin distributions, and PIN alignments. The current work adds regulation of auxin flow through PD to this theoretical framework. It provides a more comprehensive representation of the dynamic elements contributing to the developmental course of auxin strand initiation, canalization and strand interaction underlying the patterning of veins in leaves.

**Figure 8:**
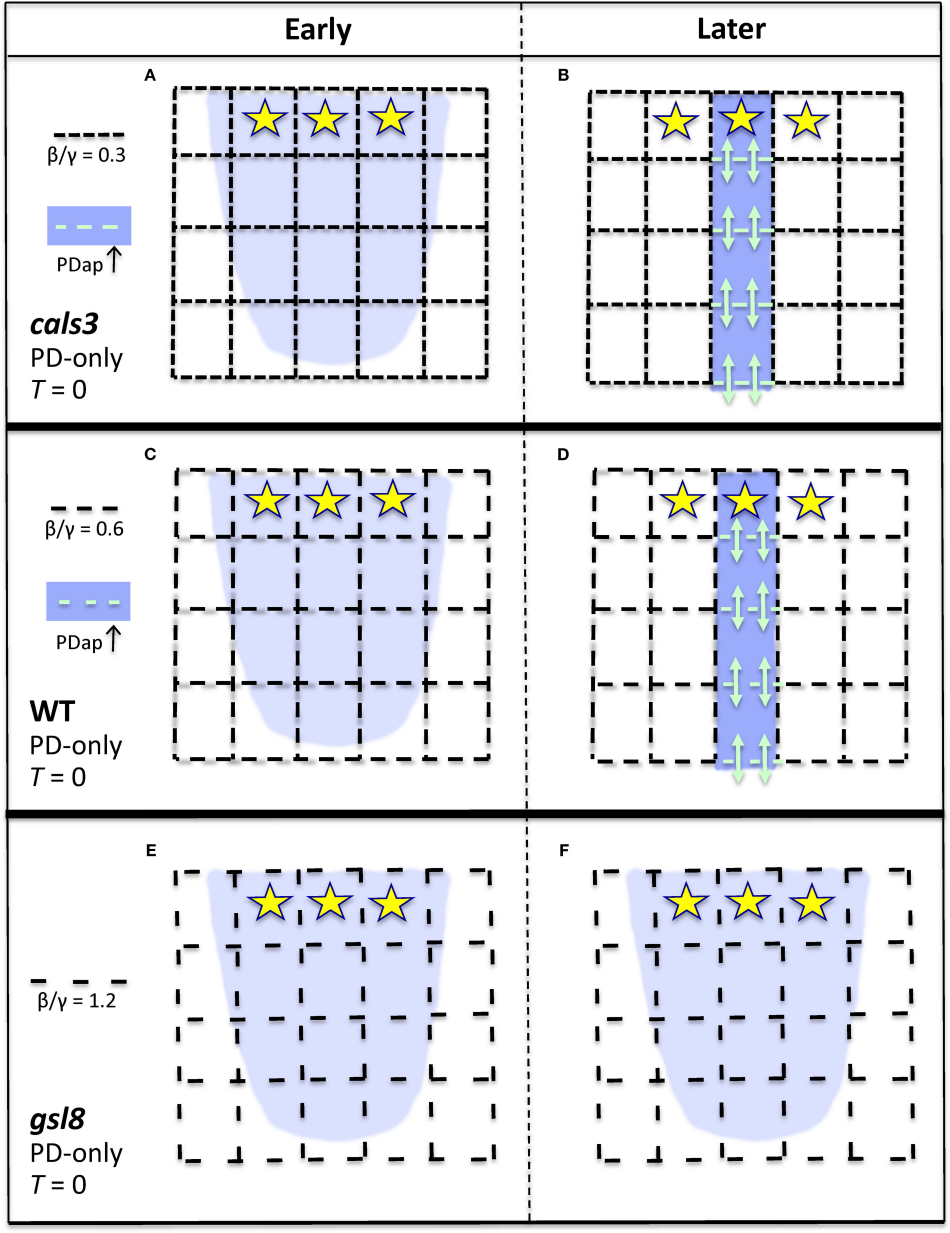
Representation of the effect of PD area on canalization, for the PD-only model (strong PIN-PAT-i conditions; **Figures 6**, **S4** results). Dash spacing in the black grid represents PD cross-sectional area between cells (*D_ij_*, Equations 1, 2). Yellow stars, auxin source; blue, auxin distribution. If background flow-independent PD area (specified by the β/γ ratio, Equation 2) is small enough, corresponding to *cals3* **(A, B)** or WT **(C, D)**, then flow-dependent regulation (α*ϕ* ^2^, Equation 2) can increase PD area in developing auxin strands (PDap, teal dashes; teal arrows indicate enhanced auxin flow through these PD), producing canalization in *cals3* **(B)** and in WT **(D)**. If the background porosity is too high, as in *gsl8*, large dash spacing in **(E)**, the flow-dependent term is not relatively strong enough to polarize the PDs (create an anisotropy) and canalize the auxin distribution **(F)**. That is, flow-dependent regulation of PD aperture is relatively ineffective in patterning if the tissue has a high intrinsic PD porosity.

The patterning of auxin into strands rather than broad distributions in strong PIN-PAT-i conditions indicates a non-PIN component of the canalization mechanism. The role of PD in this is supported by the loss of canalization in wide-PD-aperture *gsl8* mutants and by the changes in PD transport from isotropic in early stages (with lateral leakage from veins through PD into surrounding tissue) to anisotropic in later stages (with signal staying in the veins; Linh and Scarpella, 2022). Recent studies indicate that auxin signaling can play a role in regulating PD aperture: in roots, auxin activates *PDLP5*, closing PD (Sager et al., 2020); and in the hypocotyl, auxin (via *ARF7*) activates *GSL8*, which regulates callose to narrow PD aperture (Han et al., 2014). The *gsl8* phenotype in strong PIN-PAT-i conditions (Linh and Scarpella, 2022) suggests a role for this pathway in leaf vein development. In leaf vein development, though, the transition from early isotropic to the later anisotropic flow indicates a mechanism that selects for direction, favoring longitudinal in-vein PD flow over lateral out-of-vein flow. Auxin activation of *GSL8* in the nucleus cannot per se provide such a cell-scale directionality (it is scalar, when a vector quantity is needed). Mitchison's proposed role for auxin flux *ϕ* (Mitchison, 1980) can provide this anisotropic directionality. The model contains both isotropic (β/γ ratio, Equation 2, flux-independent) and anisotropic (α*ϕ* ^2^ term in Equation 2, flux-dependent) terms. This (Equation 2 plus Equation 1 with *T* = 0, i.e. the PD-only model) produces the developmental progression from early isotropic auxin distributions (**Figure 8C**) to later canalized distributions (**Figure 8D**) as flow and α*ϕ* ^2^ feedback increase. The mutant results indicate that these isotropic and anisotropic components are in a balance: for β/γ low enough relative to α*ϕ* ^2^, canalization can occur (*cals3* and WT; **Figures 8A–D**); when β/γ exceeds this (*gsl8*; **Figures 8E, F**), canalization is lost. *cals3* and *gsl8* are predicted to specifically affect non-flux dependent PD regulation: increase in a general term, such as the general permeability *D* (Equation 1), that directly influences both flux and non-flux dependent components can increase canalization (with α*ϕ* ^2^ staying relatively strong), opposite to what is observed for *gsl8*. We would also predict that very narrow PD (narrower than *cals3*), would impede or shut off vein extension. The model does not imply a particular action on lateral walls: as long as the in-vein flow becomes sufficiently high in relation to the out-of-vein flow, the vein becomes isolated from the surrounding tissue. These results are consistent with measured anisotropies of PD permeability (to fluorescein) in mature leaves, where lateral permeability from the midrib and petiole is comparable to the isotropic permeability in the stem (approx. 0.6 µm/s), and longitudinal permeability in the midrib and petiole is 5–10 times higher (Gao et al., 2020). We would predict that PD permeability measurements in leaf cells during vein formation would show similar anisotropy developing in time. Future measurements may additionally shed light on the molecular details of flux sensing or other vector quantity imparting directionality, perhaps supporting the tally molecule concept proposed for PIN flows (Cieslak et al., 2015).

The PD-only model (Equations 1; 2; *T* = 0) reproduces the observed supernumerary strands (due to lacking the CP-forming UTG-PAT, Equation 4) and slower extension of strands (due to lacking WTF-PAT; Equation 4) in PIN-PAT-i conditions compared to normal (Mattsson et al., 1999). These results are consistent with the observation that PIN-PAT-i delays progression from isotropic to anisotropic PD flows (Linh and Scarpella, 2022).

**Figure 9:**
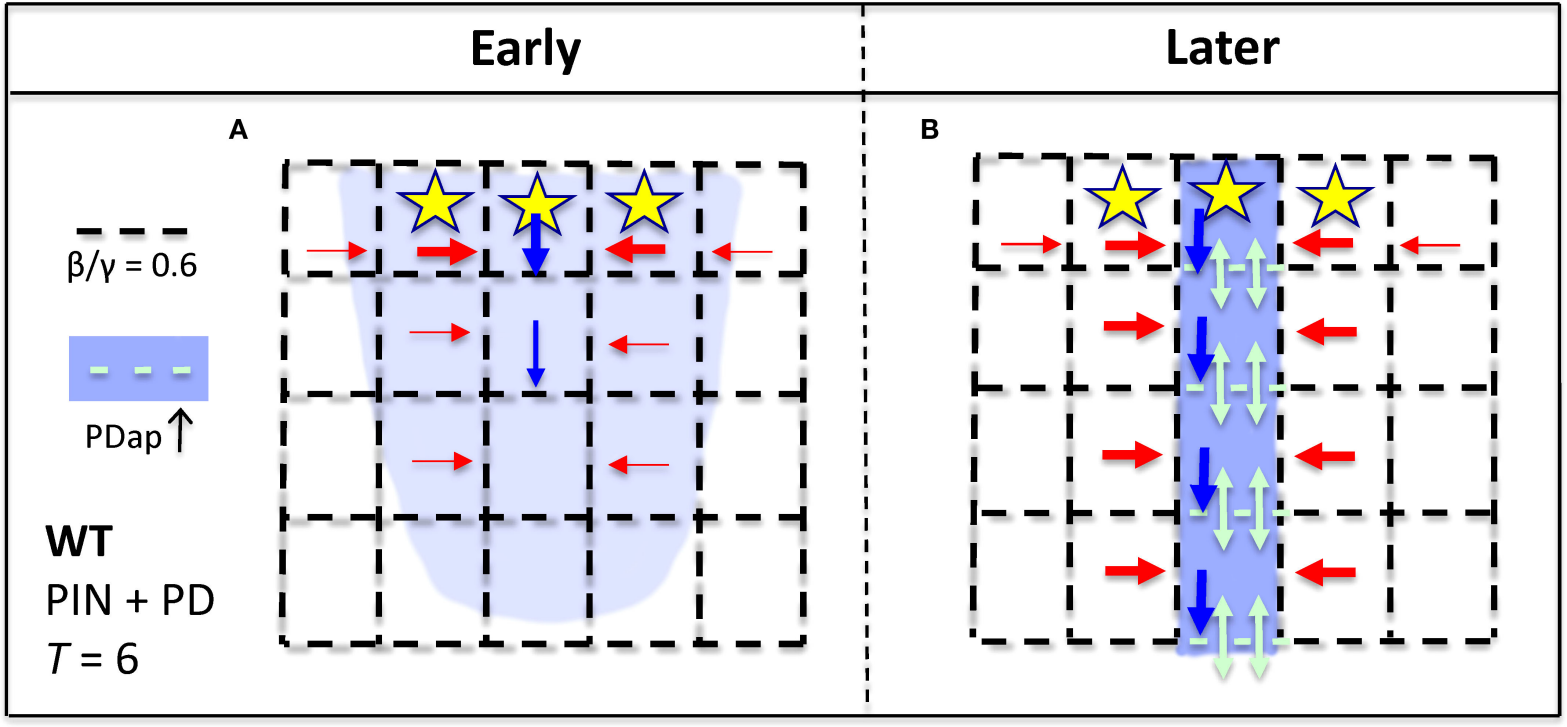
Representation of the full PIN + PD model (Equations 1–4) of normal, WT conditions. At early stages **(A)**, the auxin source (yellow stars) induces UTG (red arrows) and WTF (blue arrows) PIN allocation, generating the ‘reverse fountain’ CP; auxin also flows through intrinsic levels of PD (dash spacing in black grid; β/γ, Equation 2). Later **(B)**, both PIN and flow-dependent PD regulation (α*ϕ* ^2^ term, Equation 2; PDap, teal dashes; teal arrows indicate enhanced auxin flow through these PD) contribute to canalization of the auxin distribution (blue background in cells).

The full model (see schematic in **Figure 9**) combines PD (Equations 1, 2; black and teal, **Figure 9**) and PIN (WTF and UTG, Equations 3, 4; *T* = 6 in Equation 1) dynamics. The PD and PIN flows operate in parallel, consistent with recent results on auxin flows in the mature leaf midrib during shade response (Li et al., 2024). This generates WT patterns, including CP formation via the UTG allocation of PIN (red arrows, **Figure 9**) and rapidly extending provascular auxin strands, with WTF PIN allocation (blue arrows, **Figure 9**) reinforcing the PD in-vein flow (teal arrows, **Figure 9**). The PIN + PD mechanism also shows improved fits for mild to moderate NPA-treatment results (e.g. Mattsson et al., 1999, Mattsson et al., 2003) compared to prior PIN-only models, in particular with respect to forming extra CPs and reduced strand extension. In particular, PIN-only models lacked a canalizing mechanism as *T* was reduced or zeroed, strongly curtailing or shutting off strand formation and extension (Bayer et al., 2009; Holloway and Wenzel, 2021). Finally, the model produces extra CPs and thus more veins in response to exogenous IAA, as observed experimentally (Scarpella et al., 2006; Sawchuk et al., 2007; Verna et al., 2019; Linh and Scarpella, 2022).

### 4.2 Future considerations for the model

The model results indicate relative PD and PIN contributions to vein patterning and canalization. The permeabilities for each develop dynamically and colocalize in auxin strands. The anisotropy in the PD component is consistent with measured PD permeabilities in mature leaves (Gao et al., 2020). Future high resolution imaging of auxin flows, particularly in conditions varying PIN or PD permeability, could potentially provide new values for PIN and PD permeabilities and how they change temporally and spatially during canalization, for refining the model.

Observation of the same trends in strand formation across simulations with i) fixed size irregular, ii) growing and dividing irregular, and iii) fixed size regular cellular grids indicates robustness of the dynamics to variation in tissue geometry, wall orientations or cell area (which varies two-fold in i and ii). The irregular cell size models, particularly with growth and cell division, are more realistic representations of the developing leaf.

Experimental evidence is increasingly showing that normal vein patterning in leaves depends on a) auxin transport through PIN, b) auxin transport through PD, and c) auxin signaling within cells (see reviews by Scarpella, 2023, Scarpella, 2024). Modelling has primarily focused on a, auxin flow and PIN dynamics. (There is also scope for further investigations into other transmembrane transporters, such as the ABCB efflux proteins (e.g., Geisler et al., 2005; Geisler and Dreyer, 2024) that co-regulate with PIN (Mellor et al., 2022) or AUX1/LAX auxin influx carriers (e.g., Swarup et al., 2001; Marchant et al., 2002), particularly with pharmacological or genetic inhibition of PIN activity.) The present work has added b, auxin flow and PD dynamics. This updates the original ‘canalization hypothesis’ regarding auxin feedback on provascular strand formation (Sachs, 1978; Mitchison, 1980; Mitchison, 1981) in terms of its different components, including contributions to canalization from the UTG and WTF aspects of PIN transport and from PD transport.

The next steps in developing a quantitative representation of the leaf vein patterning process should include c, the role of auxin signaling within the cell. This, as well as consideration of auxin synthesis (or metabolic regulation of active auxin levels) within the leaf, may be particularly relevant to the formation of vein loops: auxin synthesis mutants (Kneuper et al., 2021) and interference with auxin signaling (Linh and Scarpella, 2022) both produce simple primary and secondary veins without looping (similar to the red and blue traces in **Figure 1D**, and the focus of the current model, e.g. **Figure 4A**). Triple interference with PIN transport, PD transport and auxin signaling removes the formation of organized veins (ibid). *gn* (*gnom*) mutants show a similar phenotype, indicating that *GN* is a master regulator of these three (a, b, c) aspects of leaf vein patterning and canalization (ibid). Incorporation of *GN*’s effects and auxin signaling into spatial models would clarify the role of gene regulation in the dynamic self-organization of auxin and its transport that produce the intercellular auxin flows and canalization leading to tissue-scale leaf vein patterns.

## Data Availability

The original contributions presented in the study are included in the article/**Supplementary Material**. Further inquiries can be directed to the corresponding author/s.

## Supplementary material

Supplementary Figure 1 The extending provascular strands of high auxin and PD alignments generated by the PD-only model (*T* = 0) for strong PIN-PAT-i conditions (**Figure 3**) are robust to variation in the underlying cellular arrangements. Colors and labelling as in **Figure 3**; times *t* indicated on the figures. Left column **(A, A’)**, simulations on a fixed sized irregular cellular arrangement, from **Figures 3A, B** respectively. **(B, B’)** corresponding simulations, with the same parameters, on a growing and dividing irregular cellular arrangement, from a small initial leaf shape **(B)**. Growth and division do not disrupt auxin stranding and PD patterning. **(C)** Auxin stranding (red asterisks highlight one PD track, for example) on a regular square grid. Same parameters as **(A, B)**. C' Strands extend farther with increased auxin-flux dependent PD increase (10-fold increase in α, Equation 2).

Supplementary Figure 2 The formation of normal provascular auxin patterns (PIN + PD model) and the response to a graded decrease in PIN-dependent PAT (**Figure 4** results) occur across different underlying cellular arrangements. Colors and labelling are as in **Figure 4**; same parameters in all simulations, except for *T* values noted on figures; all simulations are shown at *t* = 7 hrs. Left column **(A, D, G)** from **Figures 4A, C, D**. Normal (*T* = 6) canalized veins formed in **(A)** fixed-size, irregular cellular arrangements; **(B)** growth from a smaller initial shape (**Supplementary Figure S1B**) with cell division; and on **(C)** a regular square grid. Decreasing PIN-dependent PAT gave dose-dependent responses, with stronger effects for lower *T* parameter values. Moderate *T* decrease: **(D)** fixed-size irregular cells; **(E)** growing and dividing cells (same initial shape as **(B)**; **(F)** regular square grid. Stronger *T* decrease: **(G)** fixed-size irregular cells; **(H)** growing and dividing cells (same initial shape as **(B)**); **(I)** regular square grid. *T*-decrease effects included one or more of the following: extra CPs (vein initiation sites; red stars); decreased canalization; poorer vein directionality; and decreased vein extension.

Supplementary Figure 3 Auxin patterning responses to alterations of between-cell PD area (**Figure 5** results, full PIN + PD model) are robust to variation in cellular arrangements. Colors and labelling are as in **Figure 5**. All results are shown at *T* = 6 (normal flow through PIN) and *t* = 2 hr 30 min. Parameters are the same in all simulations, except for the β/γ ratios noted on the figures. Left column **(A, D, G)** from **Figures 5A, C, E**. Canalization is retained for *cals3* reduced-PD area (β/γ = 0.3) in fixed-size irregular **(A)**, growing and dividing irregular **(B)** (grown from **Supplementary Figure S1B**) and square regular (C) cellular arrangements (vein splitting at mid-leaf is observed in **(C)**; and also for WT PD area (β/γ = 0.6) in fixed-size irregular **(D)**, growing and dividing irregular **(E)** (grown from **Supplementary Figure S1B**) and square regular **(F)** cellular arrangements. For *gsl8* expanded-PD area simulations (β/γ = 1.2), canalization is reduced and auxin leaks into the leaf on fixed-size irregular **(G)**, growing and dividing irregular **(H)** (grown from **Supplementary Figure S1B**) and square regular **(I)** cellular arrangements.

Supplementary Figure 4 Auxin patterning for altered between-cell PD area in strong PIN-PAT-i conditions (*T* = 0; PD-only model, **Figure 6** results) is robust to variation in the underlying cellular grid. Colors and labelling are as in **Figure 6**; same parameters in all simulations, except for the β/γ ratios noted on the figures; all results are shown at *t* = 15 hrs. Left column **(A, D, G)** are from **Figures 6A, C, E**. Canalization is retained for *cals3* reduced-PD area (β/γ = 0.3) in fixed-size irregular **(A)**, growing and dividing irregular **(B)** (grown from **Supplementary Figure S1B**) and square regular **(C)** cellular arrangements; and also for WT PD area (β/γ = 0.6) in fixed-size irregular **(D)**, growing and dividing irregular **(E)** (grown from **Supplementary Figure S1B**) and square regular **(F)** cellular arrangements. For *gsl8* expanded-PD area simulations (β/γ = 1.2), PD and auxin distributions become more uniform, in fixed-size irregular **(G)**, growing and dividing irregular **(H)** (grown from **Supplementary Figure S1B**) and square regular **(I)** cellular arrangements. In **(G, H)**, some auxin stranding is still apparent; this is eliminated at higher β/γ = 4, as shown in **(G', H')**.

Supplementary Figure 5 Induction of extra veins in response to a localized boost of auxin production (**Figure 7** results; PIN + PD model; initial A_prec_ = 2 in Z1) is robust to variation in the underlying cellular grid. Colors and labelling as in **Figure 7**; same parameters in all simulations, except for the β/γ ratios noted on the figures; all results are shown at *T* = 6 and *t* = 2 hr 30 min. Left column **(A, D, G)** from **Figures 7A, C, E**. Extra veins (red stars) are induced for *cals3* in fixed-size irregular **(A)**, growing and dividing irregular **(B)** (grown from **Supplementary Figure S1B**) and square regular **(C)** cellular arrangements (the parallel veins diverge in direction mid-leaf in **(C)** and miss the central sink); and also for WT PD area (β/γ = 0.6) in fixed-size irregular **(D)**, growing and dividing irregular **(E)** (grown from **Supplementary Figure S1B**) and square regular **(F)** cellular arrangements. Extra veins are also seen with *gsl8* simulations (β/γ = 1.2), but with increased auxin leakage, in fixed-size irregular **(G)**, growing and dividing irregular **(H)** (grown from **Supplementary Figure S1B**) and square regular **(I)** cellular arrangements.
