# Supplementary material for: The role of auxin transport through plasmodesmata in leaf vein canalization and patterning
Source: Front Plant Sci. 2025 Oct 1;16:1621815. doi: 10.3389/fpls.2025.1621815 (PMC12523058; doi:10.3389/fpls.2025.1621815)
Supplement: Supplementary Figure 1 — The extending provascular strands of high auxin and PD alignments generated by the PD-only model (T = 0) for strong PIN-PAT-i conditions ( Figure 3 ) are robust to variation in the underlying cellular arrangements. Colors and labelling as in Figure 3 ; times t indicated on the figures. Left column (A, A’), simulations on a fixed sized irregular cellular arrangement, from Figures 3A, B respectively. (B, B’) corresponding simulations, with the same parameters, on a growing and dividing irregular cellular arrangement, from a small initial leaf shape (B). Growth and division do not disrupt auxin stranding and PD patterning. (C) Auxin stranding (red asterisks highlight one PD track, for example) on a regular square grid. Same parameters as (A, B). C' Strands extend farther with increased auxin-flux dependent PD increase (10-fold increase in α, Equation 2). [file Presentation1.pptx]

## Slide 1
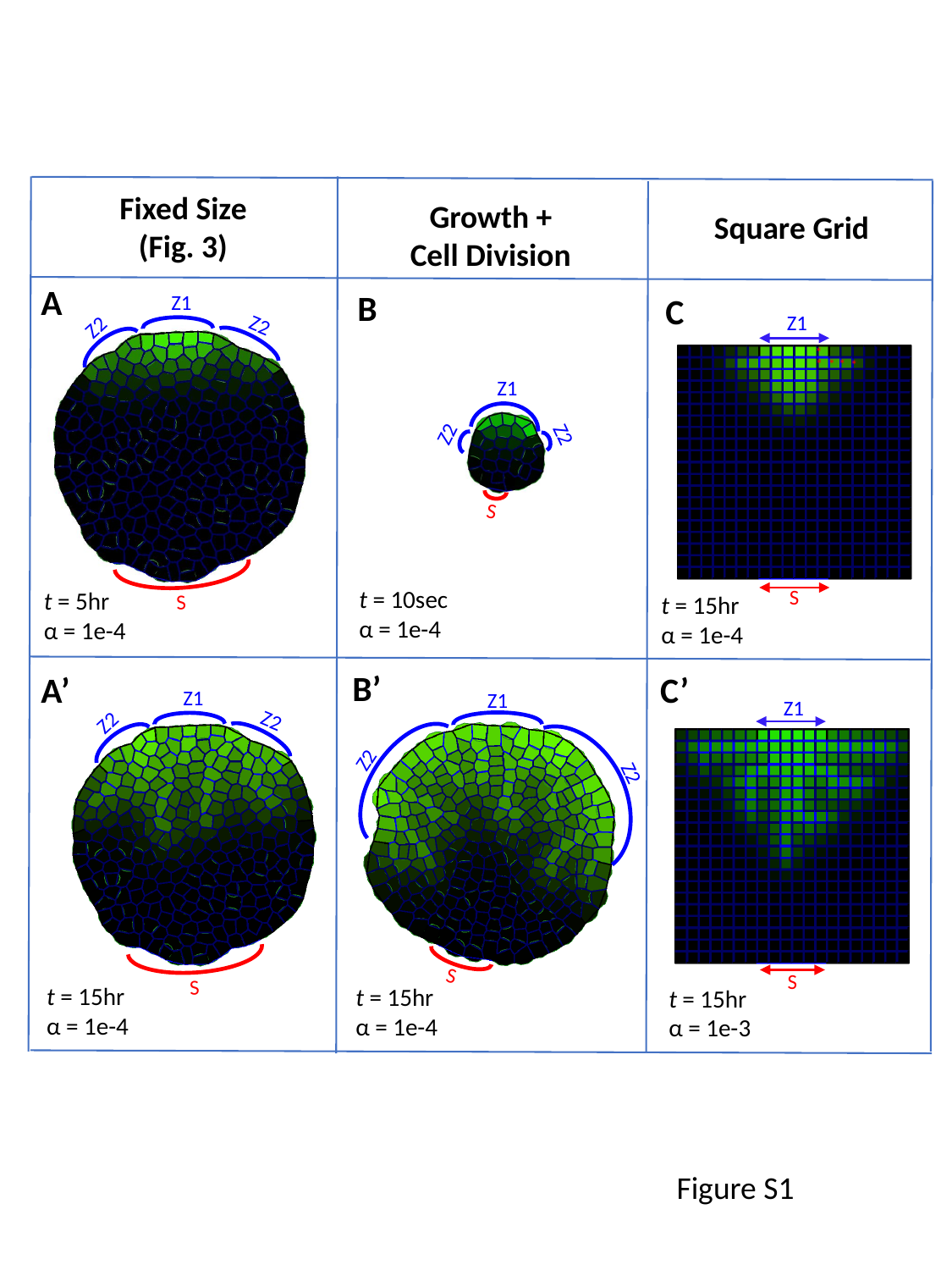

Fixed Size
(Fig. 3)
Growth +
Cell Division
Square Grid
A
Z1
Z2
Z2
S
t = 5hr
α = 1e-4
B
Z1
Z2
Z2
S
t = 10sec
α = 1e-4
C
Z1
S
*
*
*
*
*
t = 15hr
α = 1e-4
B’
Z1
Z2
Z2
S
t = 15hr
α = 1e-4
C’
Z1
S
t = 15hr
α = 1e-3
A’
Z1
Z2
Z2
S
t = 15hr
α = 1e-4
Figure S1

## Slide 2
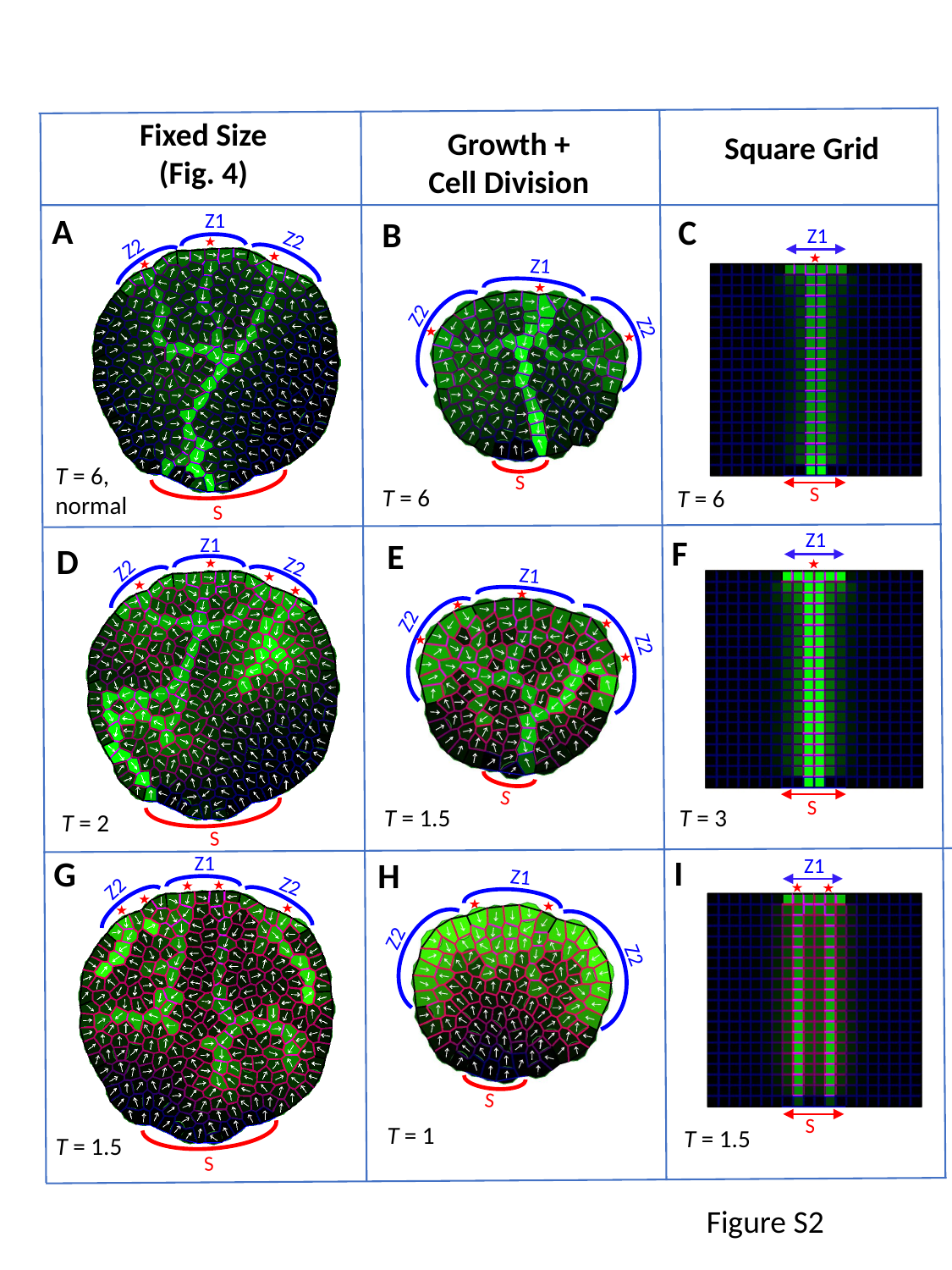

Fixed Size
(Fig. 4)
Growth +
Cell Division
Square Grid
Z1
Z2
Z2
A
S
T = 6, normal
C
Z1
S
T = 6
B
Z1
Z2
Z2
S
T = 6
Z1
F
S
T = 3
Z1
Z2
Z2
D
S
T = 2
E
Z1
Z2
Z2
S
T = 1.5
Z1
Z2
Z2
G
S
T = 1.5
I
Z1
S
T = 1.5
H
Z1
Z2
Z2
S
T = 1
Figure S2

## Slide 3
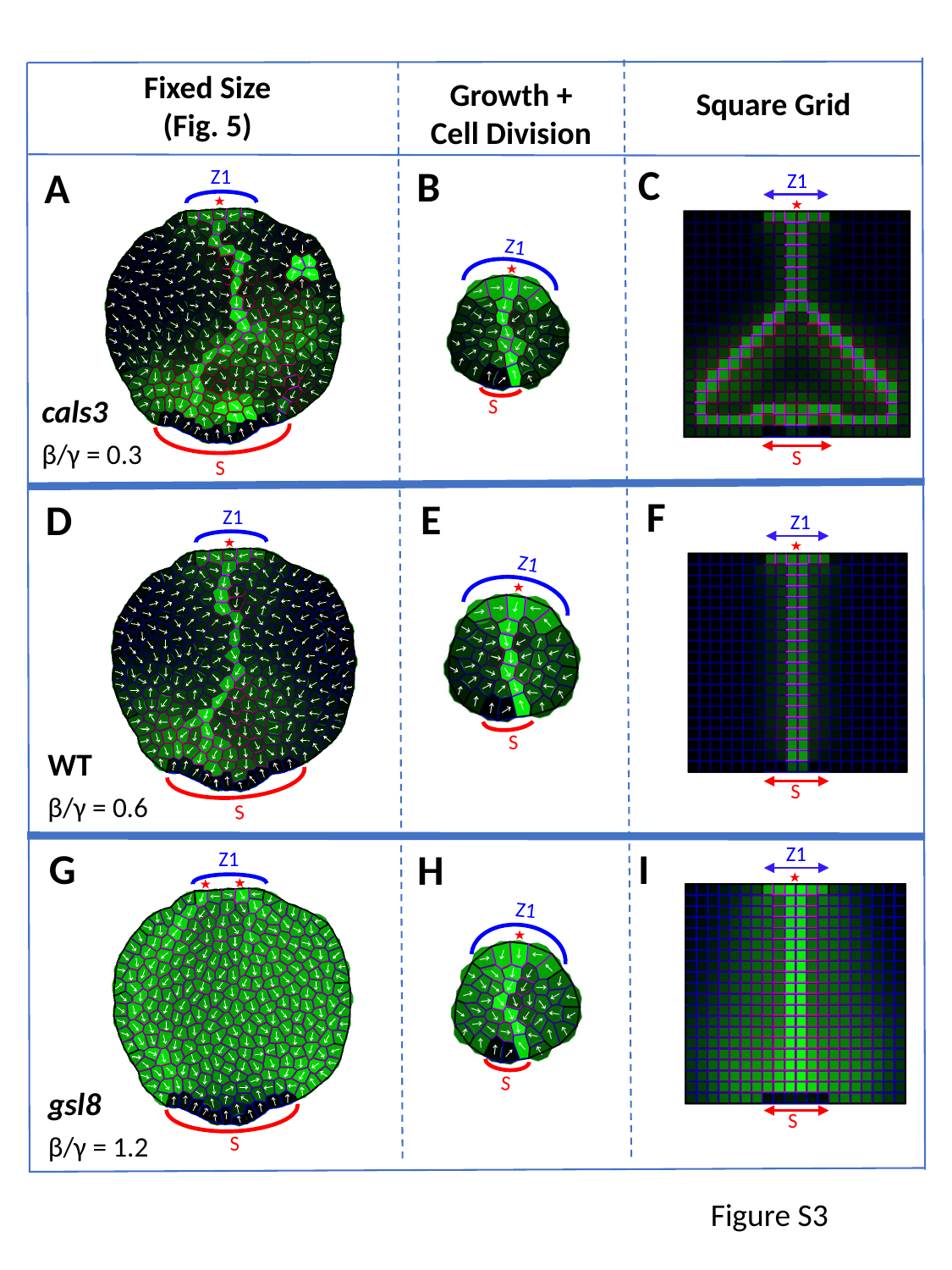

Fixed Size
(Fig. 5)
Growth +
Cell Division
Square Grid
C
Z1
S
B
Z1
S
A
Z1
S
cals3
β/γ = 0.3
F
Z1
S
E
Z1
S
D
Z1
S
WT
β/γ = 0.6
Z1
S
I
G
Z1
S
H
Z1
S
gsl8
β/γ = 1.2
Figure S3

## Slide 4
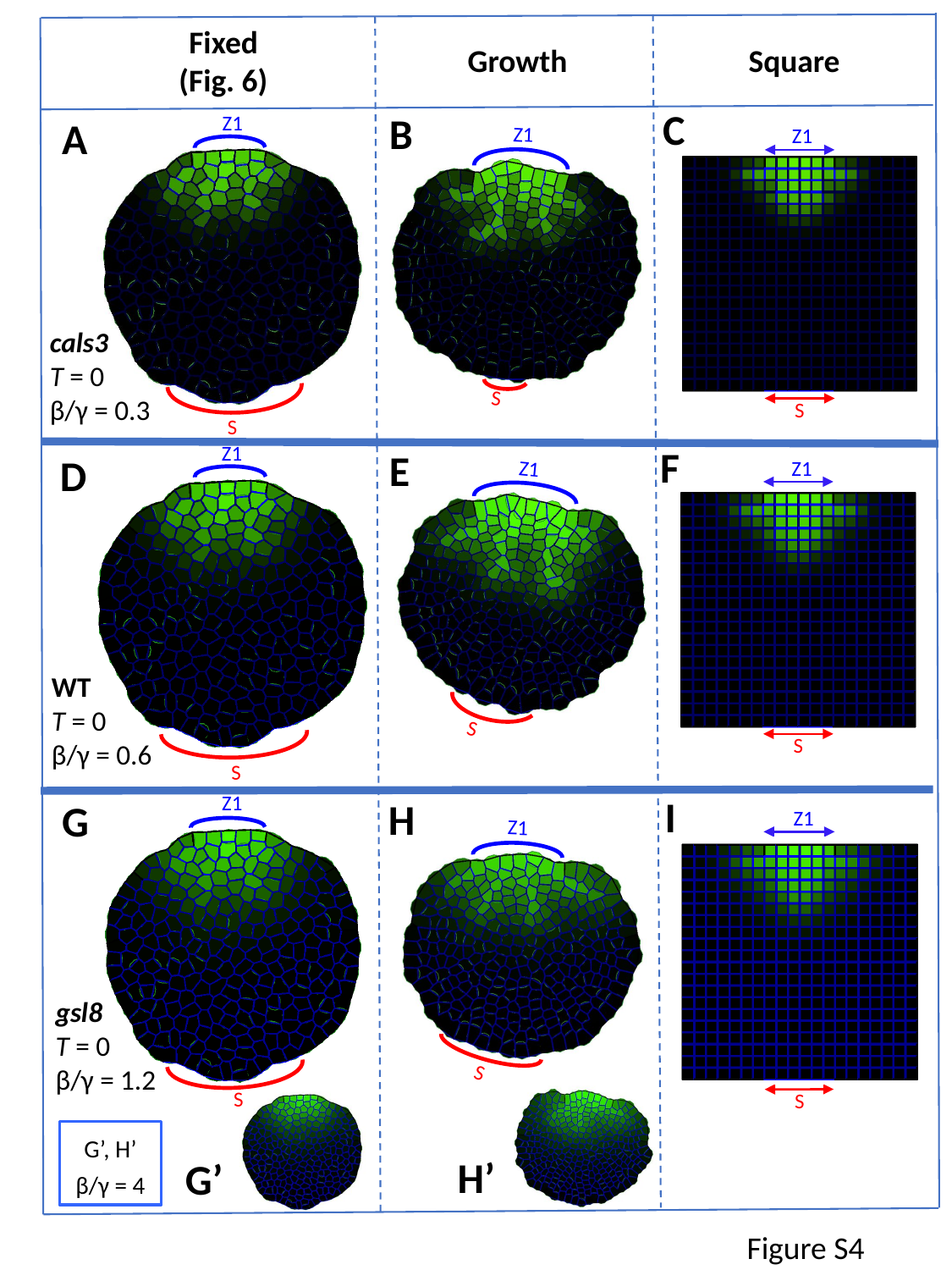

Fixed
(Fig. 6)
Square
Growth
C
B
Z1
S
A
Z1
S
Z1
S
cals3
T = 0
β/γ = 0.3
Z1
S
F
E
D
Z1
S
Z1
S
WT
T = 0
β/γ = 0.6
Z1
S
I
H
G
Z1
S
Z1
S
gsl8
T = 0
β/γ = 1.2
G’, H’
β/γ = 4
H’
G’
Figure S4

## Slide 5
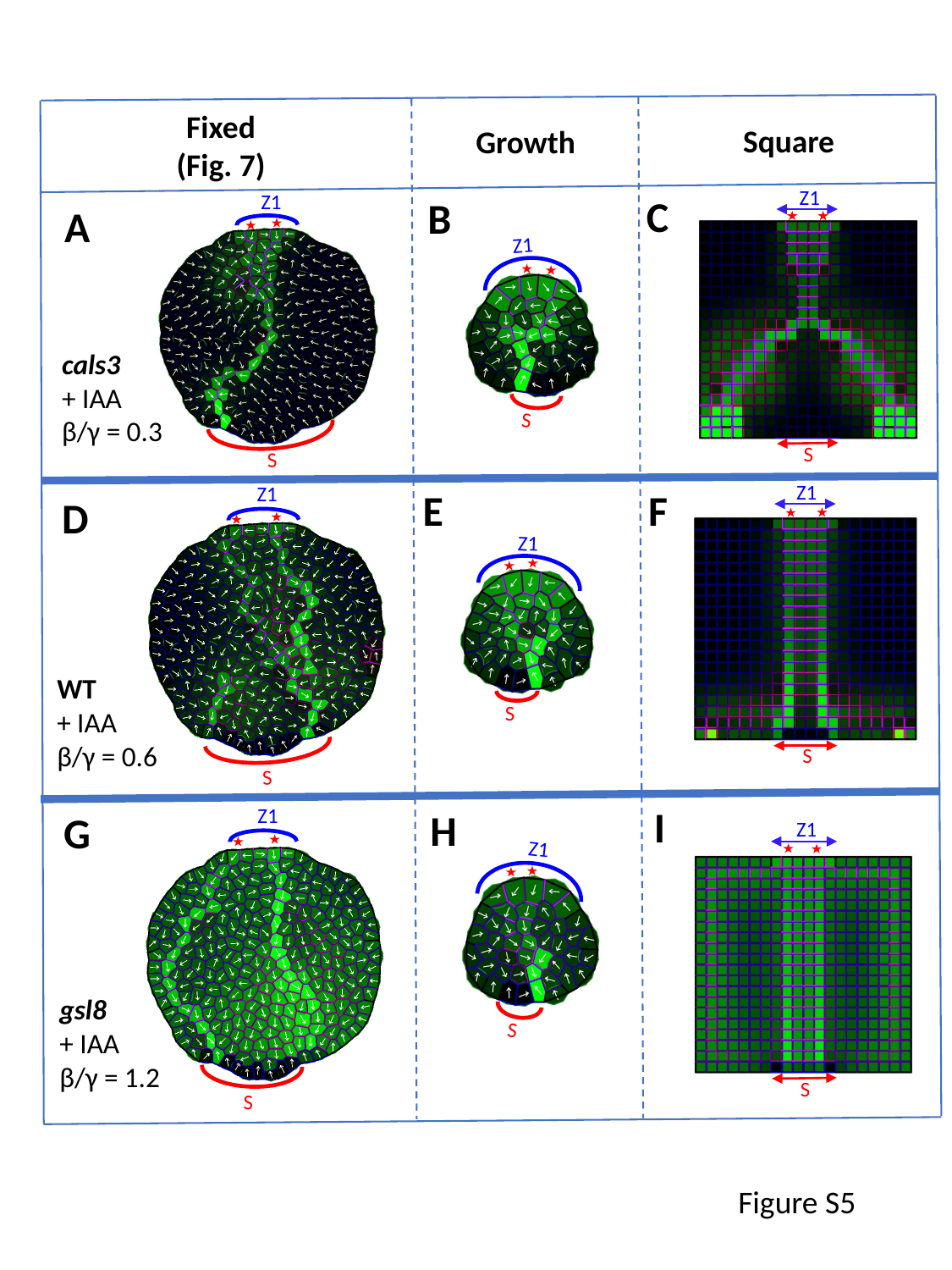

Fixed
(Fig. 7)
Square
Growth
Z1
S
Z1
S
C
B
A
Z1
S
cals3
+ IAA
β/γ = 0.3
Z1
S
Z1
S
E
F
D
Z1
S
WT
+ IAA
β/γ = 0.6
I
Z1
S
H
G
Z1
S
Z1
S
gsl8
+ IAA
β/γ = 1.2
Figure S5
